# Complex lasso: new entangled motifs in proteins

**DOI:** 10.1038/srep36895

**Published:** 2016-11-22

**Authors:** Wanda Niemyska, Pawel Dabrowski-Tumanski, Michal Kadlof, Ellinor Haglund, Piotr Sułkowski, Joanna I. Sulkowska

**Affiliations:** 1 Institute of Mathematics, University of Silesia, Bankowa 14, 40-007 Katowice, Poland; 2 Centre of New Technologies, University of Warsaw, Banacha 2c, 02-097, Warsaw, Poland; 3 Faculty of Chemistry, University of Warsaw, Pasteura 1, Warsaw, 02-093, Poland; 4 Center for Theoretical Biological Physics and Departments of Physics and Astronomy, Chemistry and Biochemistry and Cell Biology, Rice University, Houston, USA; 5 Faculty of Physics, University of Warsaw, ul. Pasteura 5, 02-093 Warsaw, Poland; 6 Walter Burke Institute for Theoretical Physics, California Institute of Technology, Pasadena, CA 91125, USA

## Abstract

We identify new entangled motifs in proteins that we call complex lassos. Lassos arise in proteins with disulfide bridges (or in proteins with amide linkages), when termini of a protein backbone pierce through an auxiliary surface of minimal area, spanned on a covalent loop. We find that as much as 18% of all proteins with disulfide bridges in a non-redundant subset of PDB form complex lassos, and classify them into six distinct geometric classes, one of which resembles supercoiling known from DNA. Based on biological classification of proteins we find that lassos are much more common in viruses, plants and fungi than in other kingdoms of life. We also discuss how changes in the oxidation/reduction potential may affect the function of proteins with lassos. Lassos and associated surfaces of minimal area provide new, interesting and possessing many potential applications geometric characteristics not only of proteins, but also of other biomolecules.

In recent years entangled proteins attracted a lot of attention and a new field of research, devoted to their studies, emerged at the interface of biophysics, chemistry, and mathematical fields of topology and knot theory. Two classes of entangled structures have been analyzed in detail to date: proteins with knots and slipknots. While for a long time it had been suspected that it is very hard to create such structures, currently more than 1000 entangled proteins are known^1^, some of them possessing quite complex knots (containing up to 6 crossings)^2^,^3^. Their properties are currently very actively studied from various experimental and theoretical perspectives.

In this work we describe a new class of entangled structures that we call complex “lassos”, which arise in proteins with cysteine bridges. We stress that lassos should not be confused with well known proteins with cysteine knots^4^, which require 3 disulfide bridges (two building the covalent loop, and the third one piercing it). It is well known that the existence of cysteine bonds is important for structure, function, and stability of proteins. For example in enzymes such as thioredoxin, cysteine bonds act as a cellular redox sensor via the oxidation status of thiol groups^5^. While cysteine bridges in general provide overall stability to proteins (for example in keratin^6^), conformational changes due to reduction or oxidation of these bonds may allow proteins to change between different functions^7^,^8^.

All the properties listed above are local, in the sense that they are related to the behavior of cysteine bridges or parts of a protein chain in the neighborhood of such bridges. In this work we show that the presence of cysteine bridges has also very interesting consequences for the global structure of proteins. Namely, we show that the presence of cysteine bridges results in very non-trivial topological configurations of the entire backbone chain that we call “lassos”, which are of biological, chemical, and mathematical interest. In particular proteins with lassos constitute a new class of proteins with the topological barrier in the free energy landscape. It is interesting to check if those proteins can fold according to the classical concept of the funnel landscape theory^9^, with cysteine bridges created in the denatured state.

One simple example of a pierced lasso in a protein with a disulfide bond has been recently reported in ref. 10, where it was referred to as a Pierced Lasso Bundle. This structure is characterized by a part of a protein backbone being threaded through a loop comprised of a part of the chain closed by a disulfide bond.

We note that structures with a similar geometric shape, called a lasso, were identified also in mini-proteins (also called lasso peptides). In this case a loop is closed by amide linkage and typically it has a size of around 10 amino acids, similarly as the segment threading this loop. The first lasso structure was identified in 1994^11^, however the first lasso peptide was the antibacterial peptide microcin J25 (MccJ25)^12^ isolated in 1992. Its lasso structure was established 11 years later along with a description of its action^13^. The peptide inhibits bacterial transcription by binding within, and obstructing, the nucleotide uptake channel of bacterial RNA polymerase. Today more than 20 such proteins are known refs 14, 15, 16, 17, 18, 19.

In this paper we show that a structure such as the Pierced Lasso Bundle, or a lasso in mini-proteins, is just a special case of a much more general and fascinating class of entangled “complex lasso” structures. In particular lassos arise in the presence of disulfide bonds and they are most common in the *α*/*β* fold of proteins. One example of a more complicated lasso structure that we identify in this work is shown schematically in Fig. 1, and an example of a protein with this configuration is shown in Fig. 2. To analyze such structures we introduced new geometric tools, based on properties of surfaces of minimal area or soap bubbles spanned on the closed loop. These tools are interesting in themselves and can be used to analyze many other entangled biomolecules, such as knotted proteins (for which they may provide new reaction coordinates to describe folding pathways), circular DNA and RNA, etc.

## Results

### Minimal surfaces and proteins with lassos

**Figure 1:**
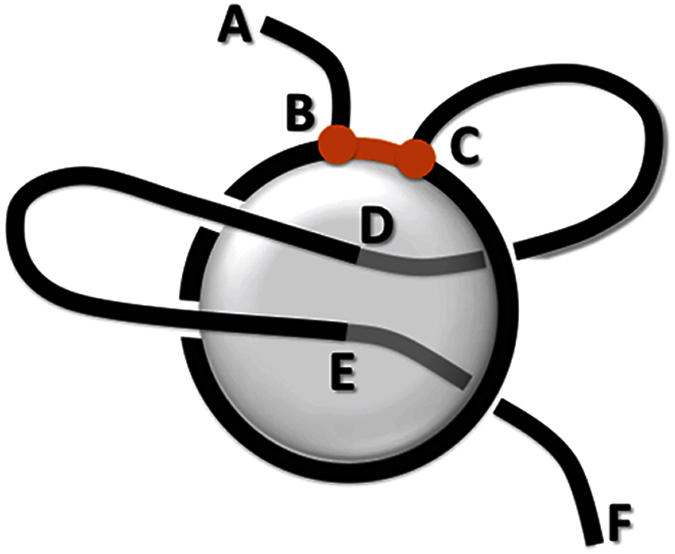
An example of a pierced lasso configuration of *L*_2_ type. Two cysteines form a disulfide bridge (orange) that closes (**B**,**C**) part of the backbone chain into a covalent loop. (**A**–**F**) Parts of the backbone chain are called tails. A minimal surface (in gray) spanned on the (**B**,**C**) loop is pierced twice by the (**C**–**F**) tail, at positions (**D**,**E**).

Let us consider a protein chain that forms a disulfide bond connecting amino acids denoted by B and C (as in Fig. 1). A part of the chain spanned between amino acids B and C (in black in Fig. 1) is called a cysteine (or covalent) loop. Denote the first and the last amino acid in the chain respectively by A and F. Parts of the chain spanned between amino acids A-B and C-F we call tails. If both tails are very short, most of the protein chain comprises a cysteine loop, and the structure has a topology of the trivial knot; this configuration is however quite rare. More often either one or both tails are long, and in favorable circumstances they pierce through the covalent loop (as e.g. in positions D and E in Fig. 1). These configurations generalize the Pierced Lasso Bundle found in a few proteins and reported in ref. 10, for which one tail is long enough to pierce once through the surface spanned on the covalent loop.

**Figure 2:**
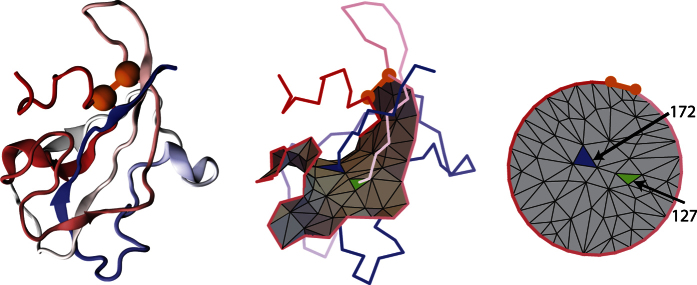
Left panel: cartoon representation of an oxidoreductase protein (PDB code 2oiz). Middle panel: triangulation of a minimal surface for 2oiz protein. The triangulated “soap bubble” surface, spanned on the covalent loop, is crossed twice by a tail, through triangles in blue and green. Two cysteines and a cysteine bond are shown in orange. Right panel: barycentric representation of a minimal triangulated surface for the protein 2oiz. Two cysteines and a disulfide bridge comprise a part of the boundary and are shown in orange. Green and blue triangles are pierced from opposite sides by 127th and 172nd tail segment respectively.

An important aspect of our analysis is how to determine whether a given tail pierces (once, or more times) a covalent loop. To this end we conducted a procedure that we call *minimal surface analysis*. First, we determine a surface of minimal area, or equivalently a surface that would be attained by a soap bubble, spanned on the covalent loop. The condition of a minimal area removes the ambiguity in a definition of such a surface. Furthermore, we consider a triangulation of this surface into small triangles. An example of such surface is shown in Fig. 2. To determine such surfaces we need to take advantage of intricate mathematical tools, also in combination with certain techniques used in computer graphics. In what follows we call such surfaces simply as *minimal surfaces*.

**Figure 3:**
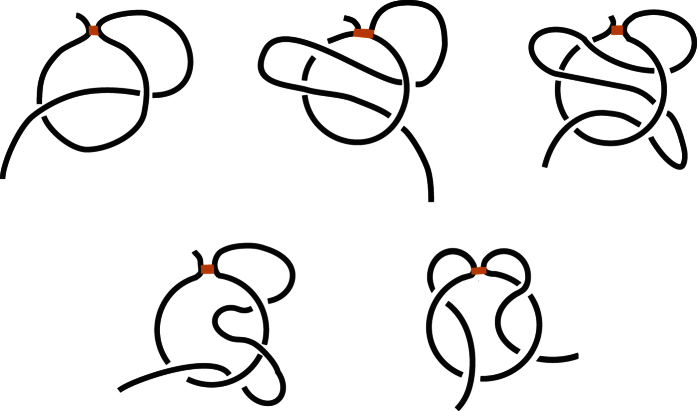
Various types of complex lasso motifs, denoted by: *L*_1_ (single lasso), *L*_2_ (double lasso), *L*_3_ (triple lasso) – top row, left to right; *LS* (supercoiling) and *LL*_1,1_ (two-sided lasso) – bottom row. More complex lassos such as *L*_6_ or *LL*_2,4_ have an analogous structure.

Once a minimal, triangulated surface is determined, it is not hard to verify whether or not it is pierced by a tail – we have to check which segment of the tail crosses one of the triangles building the surface. The simplest lasso arises when one piercing is detected. We discovered that much more complicated configurations arise in Nature that are characterized by more piercings, see Fig. 3. To understand the geometry of these configurations it is useful to project the minimal surface onto a plane using barycentric representation, and assign to triangles the numbers of tail segments they pierce – see e.g. the right panel in Fig. 2, where green and blue triangles are pierced respectively by 127th and 172nd tail segment. This representation is especially useful for more complicated configurations of a backbone, when the minimal surface may have self-intersections. We discuss more details of all these constructions in Materials and Methods.

We also stress that the minimal surface analysis can be used to analyze various properties of other (bio)polymers or their complexes. Minimal surfaces can be used to study either topological configurations (and to provide their classification), or to characterize their dynamical properties. For example, in folding processes, one could follow a shape of the nucleation site, loop formation in knotted proteins, or the mechanism of barrier crossing in the presence of a closed loop. One could also analyze evolution of minimal surfaces in reactions involving knotted circular DNA or circular RNA.

To identify proteins with lassos and to understand what function these configurations play we have conducted a thorough analysis of protein structures with cysteine loops, with sequence similarity lower than 35%. We identified a set of 2021 such non-redundant proteins in the PDB. We have found that a surprisingly large fraction of proteins, namely 18%, i.e. 376 out of 2021 protein structures with disulfide bonds, posses lasso configurations. We divided these configurations into classes which are schematically presented in Fig. 3, and which we call and denote as follows:

- **Single lasso**, *L*_1_: a covalent loop is pierced once by a tail,
- **Double lasso**, *L*_2_: a covalent loop is pierced twice by the same tail (after piercing the loop once the tail winds back and pierces it the second time from the opposite direction),
- **Triple lasso**, *L*_3_: a covalent loop is pierced three times by the same tail (similarly as in the *L*_2_ case but winding back one more time),
- **Sixfold lasso**, *L*_6_: a covalent loop is pierced six times by the same tail,
- **Supercoiling**, *LS*: one tail pierces the loop, then winds around the protein chain comprising the loop, and pierces it again (in total the covalent loop is pierced twice, each time from the same direction),
- **Two**-**sided lasso**, *LL*_*i*,*j*_: a covalent loop is pierced by two tails, *i* times by one tail and *j* times by another tail; sometimes we do not specify the numbers *i* and *j* and simply denote two-sided lassos as *LL*.

In addition, we identified a protein where lasso surface is pierced four times (PDB code 3b1b), and whose type should be denoted as *L*_4_. However, in this protein 48 amino acids have not been resolved experimentally, so we do not include it in our statistics. Nonetheless, there is no doubt that these unresolved amino acids belong to a tail that does not pierce the covalent loop, and *L*_4_ would be a type of the fully reconstructed structure.

### Classification of proteins with lassos

We have considered various classifications of proteins with lassos identified in PDB. First, we analyzed the occurrence of lasso types mentioned above in proteins, and enzymatic character of those proteins. The numbers of various lasso structures that we identified in PDB are listed in Table 1. We identified 376 proteins with lasso structure, i.e. with covalent loops pierced by tails. Among those 47 proteins have more than one pierced covalent loop (see Table S8 in Supp. Mat.), forming 16 different pierced lasso arrangements. In total we identified 433 pierced covalent loops. Most of them (331 loops in 296 proteins) are of *L*_1_ type, but we found quite many representatives of other lasso types, as shown in Table 1. In particular, we identified 14 proteins with supercoiling motif *LS*; note that in some cases the supercoiling configuration is quite easy to interpret (e.g. for PDB code: 2jh1, 4a3x), while in some other cases (e.g. PDB code: 3v83, 2jd4) the minimal surface has an intricate shape and supercoiling is less obvious to identify (see Fig. S8 in Supp. Mat.). Furthermore, among two-sided lassos we identified 8 lassos of *LL*_1,1_ type, one lasso of *LL*_1,2_ type, and one quite complicated *L*_2,4_ lasso (secreted chemokine inhibitor vCCI, PDB code 1cq3). The most complex lasso that we identified has *L*_6_ motif, i.e. its loop is pierced six times, and it is observed in Cellobiose dehydrogenase from *Myricoccum Thermophilum* (PDB code 4qi7, chain A). This protein has only one homolog (up to 30% of sequential homology, PDB code 4qi6), which is crystallized with a gap in a linker joining two domains, omitting crucial cysteine residue (which results in incorrect trivial lasso motif). Superposition of these two structures is shown in the Fig. 11 in Supp. Mat. The full list of PDB codes of proteins with various types is given in Supp. Mat. Furthermore, among all proteins with lassos in our data set we found 4 synthesized (artificial) proteins with the following PDB codes and motifs: 3eeq (*L*_3_), 1zd0 (*LS*), 2yhg and 4jgl (*LL*_1,1_ and *LL*_1,2_ respectively).

#### Lassos and function

We found that 39% of proteins with lassos are classified as enzymes. These proteins were grouped into separate classes according to the first number in Enzyme Commission classification. The largest fraction of enzymes, 22 out of 46 proteins, were identified as double lassos *L*_2_. Among single lassos (*L*_1_ type) 117 proteins out of 296 are enzymes. Proteins that posses enzymatic activity are most often identified as hydrolases and oxidoreductases, which however might be a result of high abundance of such enzymes among crystallized structures. Proteins of non-enzymatic character were grouped according to the PDB classification. These are often toxins, antimicrobial proteins, enzyme inhibitors, immune system related, or can cause adhesion of cells. The numbers of enzymes, enzyme groups, non-enzymes and non-enzyme classes among proteins with lassos are shown in Table 1. The protein class they represent is given in Table 2.

Some particular functions seem also related to lasso types. Motif *L*_1_ characterizes quite large number of binding proteins, antimicrobial, viral or immune system related proteins, transport proteins and toxins. Many proteins with *L*_2_ motif are cytokines, signaling proteins and proteins related to the immune system. Many structures with *L*_3_ motif are transport proteins. There are not so many examples of supercoiling and *LL* structures so it is hard to provide statistics, however adhesion proteins seem to appear quite often in those groups.

We identified lasso configurations among some classes of proteins whose biological function could be supported by complex topology. In particular we found lasso geometry in carbonic anhydrases of PDB codes 3q31 (of *L*_1_ type), and 3iai, 4g7a, 4ht2 (of *LS* type). These are proteins with very shallow knots (which could be untied by thermal fluctuations) that partially form active sites; stability of those knots could be explained by the existence of the lasso. Lasso is also found in the following membrane proteins: 2ydv, 4gwn, 4io2 (of *L*_1_ type), 3nsj (*L*_3_), 3v83 (*LS*). Here, similarly to knotted proteins, lasso loop could strap together functionally important helices that pierce a lasso minimal surface. Those observations suggest that lasso configurations provide global stability to proteins, without blocking internal motions necessary to perform biological functions.

While 39% of enzymes among proteins with lassos is a substantial fraction, note that it is much less than in the case of knotted proteins, whose majority is classified as enzymes^1^. To investigate the correlation with function further, we searched for post-translationally modified residues in lasso proteins. We found four pierced loops of L1 type containing such modified residues (Table 9 in Supp. Mat.), and in 10 other structures modified residues were located externally to the covalent loop (Table 10 in Supp. Mat.). However, we have not observed any direct correlation between the lasso type, function, location of piercing, and location of the modified residue.

#### Organism classification

Let us now analyze how often disulfide bridges and lassos arise in various kingdoms of life. 376 identified proteins with lassos comprises 18% of the nonredunant set of proteins with disulfide bridges identified in all kingdoms (2021 structures). In Table 3 we present how many proteins with disulfide bridges are identified in various kingdoms, and how often lassos appear in these kingdoms. Lassos arise most often in viruses, fungi and plants, where we identified them in around 25% of all proteins with disulfide bridges in a given kingdom (in viruses a majority of them are hemagglutinins or spike proteins; note that even though viruses are represented only by 24 structures, lassos come from at least 15 different viruses). Lassos also appear quite often in animals, in 20.4% of proteins with disulfide bridges. In bacteria, archaea and protistae they are found in around 10–15% of cases. It would be desirable to understand this statistics in more detail, especially the abundance of lassos in viruses, plants and fungi.

### Folds and secondary structure of lassos

Let us discuss now the secondary structures and protein folds that appear in lassos, by considering two main classifications from CATH: Class and Architecture^20^. Class describes secondary content of proteins, which can be classified as *mainly α, mainly β, α*/*β*, or *Few Secondary Structures*. Architecture describes general shape/fold of proteins. Here we discuss the most important features of *L*_1_, *L*_2_ and *L*_3_ motifs; for detailed analysis see Table 11 in Supp. Mat.

#### Triple lasso

**Figure 4:**
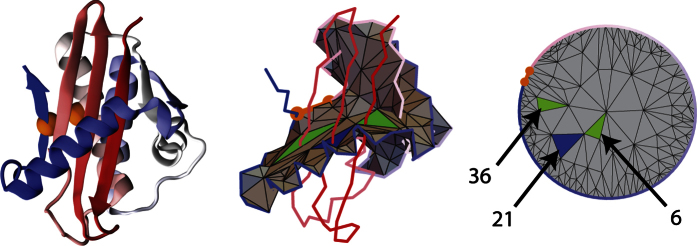
Representation of hydrolase protein (PDB code 2ehg). Left panel: cartoon representation of hydrolase protein (PDB code 2ehg). Middle panel: triangulation of a minimal surface for 2ehg protein. The triangulated “soap bubble” surface, spanned on the covalent loop, is crossed three times, through triangles in blue (once) and green (twice). Two cysteines and a cysteine bond are shown in orange. Right panel: barycentric representation of a minimal triangulated surface for 2ehg protein. Two cysteines and a cysteine bond comprising a part of the boundary and are shown in orange. Green and blue triangles are pierced from opposite sides by 6th, 21st and 36th tail segment.

We identified this motif in 25 proteins, out of which 16 are classified as *mainly β* and posses *β barrel* fold. Only four proteins are of type *α*/*β*, see example in Fig. 4. Detailed analysis of all proteins with a *L*_3_ motif shows that the minimal surface in 95% of the cases is crossed either by: (A) a well defined *β* hairpin and *β* strand, or (B) by *β* strands with an unstructured straight segment, or (C) by parallel *α* helices (in the case of helical proteins). For example in protein 1 RNase H from a *Hyperthermophilic Archaeon* with Double-stranded RNA-dependent RNase activity (PDB code 1u3d), the surface of a loop is formed by a well packed *α* helix, and it is crossed by three *β* strands. Here the *L*_3_ motif could be responsible for a hyperthermophilic stability of this protein.

#### Double lasso

We found that (similarly as in *L*_3_ case) 95% of proteins with *L*_2_ motif are of *mainly β* type, forming two types of architecture: *β barrel* or *sandwich* fold. Only three proteins form *mostly α* configuration. Similarly as for *L*_3_, the geometry of the *β* hairpin or its disordered turn form a structure that crosses the minimal surface. We found only two exceptions (PDB codes: 4psc, 2x97), where the loop is too big to constrain the protein chain to a particular shape. It is worth mentioning that Thymus and Activation-Regulated Chemokine (TARC) protein, whose loops are made of 23 amino acids, belongs also to *L*_2_ class. We found that this protein has a very tight conformation, which is stabilized by almost disordered but closed loop that stabilizes the *L*_2_ motif by antiparallel *β* strands. We identified a similar conformation in four secretory proteins.

#### Single lasso

The *L*_1_ motif is found in 296 proteins and for 189 structures CATH classification is available. These proteins are classified in 39% of cases as *mainly β*, in 47% as *α*/*β*, in 9% as *mainly α*, and in 5% as *Few Secondary Structures* (the structure of those proteins was determined mostly by NMR techniques). The existence of a lasso motif in proteins that posses very small number of stable secondary structure elements or just disordered loop class *Few Secondary Structures* strongly suggests that *L*_1_ motif could be responsible for striping structure together. Some of those proteins are members of the Kunitz-type serine protease inhibitors family, where it was shown that disulfide bond imposes high stability^21^. Existence of topological constraints additionally explains high stability of these proteins, not only to protect it against high pH, but also to stabilize them in high temperature. The second class of proteins, *mainly α*, is represented by two folds: helical bundle and orthogonal bundle. The helical bundle was identified in particular in the first example of a lasso in leptin^10^. Here we found that helical bundle is also observed in human group (hGX), which is secreted phospholipase A2 (sPLA2).

### Mini-proteins

Our method can be also applied to mini-proteins (lasso peptides) with a lasso configuration *L*_1_ (in our notation). We have not included those proteins in the statistics discussed above, because for mini-proteins a loop is formed by amide linkage (between free N-terminal amine group of glycine or cysteine and carboxylic group in side chain of glutamic acid), although some mini-proteins (class I and III^14^) contain also disulphide bonds. The loops formed by these bonds are however trivial from our point of view (class *L*_0_). Nonetheless, we can also use our techniques of minimal surfaces to study mini-proteins. In this way we can reveal a new piece of information, i.e. we can identify which amino acid pierces the lasso loop. Such an amino acid is commonly called a “plug”, and it blocks a tail from piercing further through the lasso loop. We provide a detailed description of all known lasso proteins, with this new information (identification of the plug amino acids) included, in Table 13 in Supp. Mat.

## Discussion and Conclusions

In this work we have identified new entangled motifs that we called lassos. They may appear in (bio)polymers that form loops, through which some part of a backbone chain is threaded. While proteins with disulfide bridges (that define covalent loops) have been the object of our analysis, such motifs could be also identified and analyzed in other structures (e.g. mini-proteins, circular DNA or RNA). Moreover, the loop can be closed also by a virtual bond joining spatially nearby residues^22^,^23^. Such approach can be useful in studying e.g. knotted or slipknotted proteins. Its versatility makes it more general than some other methods; for example it can be used instead of the KMT method^24^ in the analysis of entangled structures.

To identify a lasso motif unambiguously we proposed to consider a surface of minimal area spanned on a (covalent) loop. We classified lassos according to the number of times and directions from which this surface is pierced by tail segments. In proteins we identified six lasso motifs, with a covalent surface pierced once, twice, three times, or six times, supercoiling, and a surface pierced by two tails. We discussed various classifications of proteins with lassos (their presence in various kingdoms of life, enzymatic character, etc.), as well as their secondary structure, fold types that they form and possible function.

It is interesting to note that analyzing all proteins deposited in the PDB we have identified six lasso types described above, but not any more complex geometric structures. This implies that the set of lasso types identified by us is unique and a substitution of a single amino acid to form a cysteine bridge does not introduce new topological motifs. Moreover, because cysteine bridges are most strongly conserved type of interactions^25^,^26^ (a single amino acid is conserved in around 50%), we expect that complex lasso topology is conserved to provide advantage to a hosting organism. It would be interesting to study in more detail how the presence of complex lassos affects function or stability of proteins.

Stability of complex lasso structures may be induced by many factors. In structures with small loops, the stability may be a result of steric hindrance, similarly as in mini-proteins (class II). In larger systems it is possible, that the steric hindrance of the piercing chain would be insufficient to keep the chain in its native position. In such case, probably the interaction preserving the general tertiary structure may be involved in maintaining the lasso type. To identify such interactions, we calculated the statistics of occurrence of amino acids in the spatial proximity of the piercing. We have not observed any particular type of amino acids for larger loops. However, for small covalent loops (up to 30 residues) the piercing residue is always surrounded by at least one bulky amino acid, located up to 5 residues from piercing (see Table 12, Supp. Mat.). This correlation suggests that small, unstructured proteins are stabilized via steric hindrance.

We also note that proteins with lassos provide a unique opportunity to study the free energy landscape – threading a tail through a covalent loop results in a well-defined topological barrier (or several barriers for more complicated lassos), whose properties and topology can be analyzed in detail experimentally. It should be possible to reversibly fold such configurations in reducing conditions (when disulfide bridge is unstable), however under oxidating conditions folding could be hindered by topological constraints. Disulfide bonds can be reduced or formed in reducing or oxidating conditions that also affects the free energy landscape. It was shown that stable intermediate states with disulfide bonds serve as a template to drive remaining chain of a protein into a more compact conformation, allowing subsequent interactions to complete the final stages of folding^27^. Investigation of the folding mechanism of lasso proteins provides a unique opportunity and should shed light on folding of knotted proteins *in vitro*, which is still not fully understood even theoretically^28^,^29^.

Proteins with disulfide bridges, e.g. cysteine knot mini-proteins also known as knottins, are an attractive class of agents for the development of peptide-based pharmaceuticals^30^. Many natural mini-proteins already possess interesting pharmacological properties that can be used as a starting point for further developments by protein engineering. Actually, the similarity to mini-proteins can give a clue for the function of *L*_1_ lasso motif, at least in case of antimicrobial (disulfide based) lasso proteins – for example this motif might serve as a “molecular plug”, as in case of the action of mini-proteins, blocking the uptake channel^13^,^31^.

The first engineered knottin was already successfully applied for tumor imaging. The cyclotides^32^, a family of mini-proteins that contain a head-to-tail cyclized backbone, also have a diverse range of biological activities, including uterotonic, anti-HIV, antitumor, and antimicrobial, although their natural function is likely that of defending their host plants from pathogens and pests. Those proteins are exceptionally stable and resistant to denaturation via thermal, chemical, or enzymatic treatments.

Identified here new topological aspect – lasso topology – provides a new tool to manipulate those proteins. Undoubtedly this tool is very useful not only in the analysis of mini-proteins, but also for all other proteins with much more complicated motifs, that we identified in this work. We hope that lasso motifs may find new therapeutic applications also beyond mini-proteins. The existence of a pierced covalent loop, which is the common feature of complex lasso proteins, mini-proteins and cysteine knots, suggests that such a configuration should have functional advantages. Studies aimed in revealing the functional implications of such motif can be a major step towards understanding correlations between geometry and function.

Considering possible functions of lasso proteins it is interesting to compare them to their chemical relatives – rotaxanes. From the chemical point of view, the *L*_1_ lasso configuration is the [1]rotaxane structure. In rotaxanes, the surrounding cyclic compound was shown to induce additional enzymatic stability^33^ or to modify the fluorescence^34^ of the piercing compound. Rotaxanes in which the surrounding ring has two different stable positions around the piercing element were used in nanomachinery^35^,^36^ or as a molecular electronic memory^37^. Discovering the lasso protein with bistable covalent loop could give researchers the evolutionary-optimized tool for further development of the rotaxane applications. On the other hand, there is no exact analogs of the *L*_2_, *L*_3_, *L*_6_, LS and LL lasso types. Most similar, doubly-threaded [3]rotaxane are still the exotics of chemical topology^38^,^39^,^40^,^41^,^42^ with only one, recent example of triply-threaded [4]rotaxane known to us^43^. Properties and use of such compounds are still poorly understood, and hard to correlate with lasso proteins.

It is also important to understand how the formation or breaking of cysteine bonds, in consequence of changes in the oxidation/reduction potential, may affect the geometry and complexity of lasso structures. Such processes should impact, and possibly turn on/off, biological functions of proteins with lassos. We hope that in fact one could even steer such biological functions by changes in lasso geometry, introduced by modification of the oxidation/reduction potential.

Complex lasso motifs also provide a new interesting object of investigations for soft condensed matter studies. Probability of the appearance of different lasso structures under different solvent condition, a size of the lasso loop, the length of its tails, and other aspects of lasso geometry undoubtedly deserve further studies. Our analysis also shows that a single chain can accommodate a few lassos (Table 8 in Supp. Mat.). It is interesting to analyze how they can be located along the sequence and possibly linked. Yet another interesting question is whether one can define lasso motifs e.g. in quaternary structures, i.e. are there covalent loops formed by several chains, which are pierced by another chain. A list of multimeric proteins with at least one pierced lasso is shown in Supp. Mat. (5th section) and it would be interesting to analyze their properties. We plan to address some of these issues in future work.

Finally we note, that based on the tools described in this paper, a systematic review of the whole PDB has already been conducted and all identified proteins with lasso motifs (including those presented in this paper) are deposited in the LassoProt database^44^. We are convinced that analysis of all those structures and their lasso motifs deserves further thorough studies.

## Materials and Methods

### Protein dataset

In this work we identified 2021 proteins with covalent loops, from among a list of non-redundant PDB entries determined by the PICES server as August 2016, with the following parameters: 35% of sequence similarity, including X-ray, NMR, CEM structures and proteins with unresolved parts. From the resulting dataset we retained all proteins with disulfide bridges, for which a covalent loop was comprised of more than 10 amino acids.

For protein structures with unresolved parts, we reconstructed their chains by our package based on Modeller^45^. In the modeling procedure missing fragments were reconstructed based on homological structure. In case a homological structure is not known and the number of missing atoms was smaller than 10 amino acids, we used the Modeller loop prediction method. In the dataset we also included leptin considered already in ref. 10, for which 14 unresolved C_*α*_ atoms were reconstructed following^46^. Other protein structures with unresolved parts were excluded from our set.

The above analysis resulted in a total of 2021 protein chains with covalent loops, with the shortest loop comprised of 13 amino acids. Among those, conducting the minimal surface analysis described below, we identified 376 proteins with pierced loops (lassos structures).

### Minimal surface analysis

We propose to classify lasso configurations by the number of crossings of backbone tails through the surface spanned on the covalent loop. A crucial aspect in identification of a lasso type is a well-defined construction of such surfaces. We propose to consider surfaces of minimal area, that we call *minimal surfaces*. There are several equivalent definitions of such surfaces – for example, they can also be characterized as possessing zero mean curvature; surfaces of these shapes are attained in particular by soap bubbles. Despite these rather simple characterizations, quite an intricate mathematical apparatus (involving variational calculus, isoperimetric problems, etc.) is necessary to describe these surfaces. Furthermore, in practical applications, we need to work with discrete (triangulated) versions of such surfaces. There are several algorithms, used in particular in computer graphics, that allow to determine triangulations of minimal surfaces. In our work we implemented an algorithm discussed in ref. 47. The initial data in this algorithm consists of coordinates of amino acids in the covalent loop. In the first step we choose some arbitrary triangulation involving *N* vertices, which will be transformed into the minimal triangulated surface. These *N* vertices can be chosen somehow arbitrarily; one simple idea is to consider triangles made of pairs of consecutive amino acids in the covalent loop and the center of mass of the loop; the center of mass of each such triangle can be chosen as one of those *N* starting vertices (and segments that join the center of each triangle with its vertices become parts of the triangulation under construction). If the number of such triangles is smaller than *N* (which is typically the case if we want to construct better approximation to the smooth minimal surface), we can subdivide each such triangle into 3 smaller triangles, choose their centers, and repeat this procedure unless a set of *N* points (together with a triangular web that follows from the above procedure) is specified. In our analysis we used a variation of this method, which is described in more detail in Supp. Mat.

Once this data is specified, we perform two operations: first, positions of all *N* vertices are adjusted to decrease area locally around each vertex (i.e. a position of a given vertex is adjusted based on locations of all vertices it is connected to). Second, we consider all pairs of triangles of vertices *a, b, c* (one triangle) and *b, c, d* (another triangle) that share the edge *bc*, and – if this would result in triangulation of smaller area – replace this edge by the edge *ad*, thereby redefining the triangulation. Repeating this sequence of operations many times leads to a triangulated minimal surface (more precisely, this may lead only to a local minimum, however in our practical applications we have not come across any surfaces that might seem inappropriate). Varying the initially chosen number of points *N* allows to adjust the size of meshes in the resulting triangulated surface; for larger *N*, the final triangulation provides better approximation to a smooth minimal surface, however it may take more time to determine it. Depending on the nature of a problem (e.g. determining just a single surface, or a large set of surfaces for many proteins, or for time series of configurations) *N* may be adjusted to provide an optimal computational time.

Once the minimal triangulated surface is determined, we can identify a lasso type by identifying in which direction and by which segments of the protein tail (or two tails) the surface is pierced. We specify a direction of crossing by a sign (plus or minus) assigned to a segment piercing the surface. To identify only well-defined lassos we imposed a condition that – in case the surface is not exceedingly bent (for details see Supp. Mat.) – there must be at least 10 amino acids between consecutive crossings with opposite signs, i.e. a piece of a tail piercing a surface is sufficiently “deep” (see e.g. the segment DE in Fig. 1).

We also demand that the segment between a disulfide bridge and the first piercing consists of at least 3 amino acids (see e.g. the segment CD in Fig. 1).

To reveal the structure of a lasso and the pattern of piercing – especially if it is hard to identify it by a naked eye – it is convenient to present the triangulated surface as a planar barycentric embedding, in which each vertex of a triangulation is an average of vertices it is connected to. By a theorem by Tutte, such representation can be uniquely determined purely from the connectivity structure of a triangulated surface. We use a well known algorithm by Tutte to determine such baricentric representation. Identifying triangles pierced by a tail in such representation and assigning to these triangles the numbers of piercing segments is very useful in various analysis. Examples of barycentric representations for proteins 2oiz or 2ehg are shown in right panels in Figs 2 and 4.

Molecular graphics and analyses were performed with the UCSF Chimera package and VMD software^48^,^49^. Chimera is developed by the Resource for Biocomputing, Visualization, and Informatics at the University of California, San Francisco (supported by NIGMS P41-GM103311).

## Additional Information

**How to cite this article**: Niemyska, W. *et al*. Complex lasso: new entangled motifs in proteins. *Sci. Rep.* **6**, 36895; doi: 10.1038/srep36895 (2016).

**Publisher's note**: Springer Nature remains neutral with regard to jurisdictional claims in published maps and institutional affiliations.

## Supplementary Material

Supplementary Information

## Figures and Tables

**Table 1:** Classification of lasso structures.

Lasso type	A	B	C	D	E
Single, *L*_1_	296	5	117	37	179
Double, *L*_2_	46	3	22	9	24
Triple, *L*_3_	25	3	4	8	21
Sixfold, *L*_6_	1	1	1	0	0
Supercoiling, *LS*	14	2	4	5	10
Two-sided, *LL*	10	1	2	6	8
Total	376	5	148	41	228

A – Number of proteins; B – number of enzyme groups with respect to the first number in EC classification; C – number of enzymes; D – number of non-enzyme classes with respect to PDB database; E – number of non-enzymes.

**Table 2:** Families of proteins with various lasso types.

Lasso	Protein families
*L*_1_	• hydrolases (85), transferases (15), oxidoreductases (14), lyases (3), isomerase (1);
	• binding protein (26), antimicrobial proteins (16), viral proteins (15), immune system related (12), transport proteins (12), toxines (11), cytokines (10), membrane proteins (9) …
*L*_2_	• hydrolases (9), oxidoreductases (9), transferases (4);
	• cytokines (11), immune system related (3), signaling proteins (3), viral proteins (3), other (4);
*L*_3_	• hydrolases (2), isomerases (1), oxidoreductases (1);
	• transport proteins (10), allergens (3), immune system related (2), viral proteins (2), other (2);
*L*_6_	• oxidoreductase (1);
*LS*	• lyases (3), hydrolases (1);
	• cell adhesion related (5), metal binding protein (2), structural proteins (1), transport protein (1), GAS(1);
*LL*	• hydrolases (2);
	• cell adhesion related (2), membrane proteins (2), toxin (1), structural protein (1), cytokine (1), transport protein (1);

In the first bullet groups of enzymes are listed in order of decreasing number of occurrences; in the second bullet PDB classes of non-enzymatic proteins are listed in order of decreasing number of occurrences. In case of *L*_1_ lasso only groups with more than 9 elements are listed.

**Table 3:** Number of proteins with disulfide bridges in various kingdoms of life.

Kingdom	#proteins ~ %	#of lassos ~ %
Animal	962~47.6%	196~20.4%
Bacteria	515~25.5%	61~11.8%
Fungus	155~7.7%	39~25.2%
Plant	147~7.3%	36~24.5%
Archaea	88~4.4%	10~11.4%
Virus	87~4.3%	24~27.6%
Protistae	67~3.3%	10~14.9%

Middle column: the number of lassos and its percentage among all 2021 proteins with disulfide bridges. Right column: The number of lassos in various kingdoms, and its percentage among all proteins with disulfide bridges in this kingdom.
