# Supplementary material for: Complex lasso: new entangled motifs in proteins
Source: Sci Rep. 2016 Nov 22;6:36895. doi: 10.1038/srep36895 (PMC5118788; doi:10.1038/srep36895)
Supplement: Supplementary Information [file srep36895-s1.pdf]

Complex lasso: new entangled motifs in proteins

Supplementary Material

Wanda Niemyska, Pawel Dabrowski-Tumanski, Michal Kadlof, Ellinor Haglund,
Piotr Sułkowski and Joanna I. Sulkowska

Contents

1 Construction of minimal surfaces 1
- 1.1 Area Minimizing . . . 3
- 1.2 Laplacian Fairing . . . 3
- 1.3 Edge Swapping . . . 4
- 1.4 Initialization . . . 5
- 1.5 Identification of lasso types . . . 5

2 Details of protein reconstruction 7

3 Full list of proteins 10

4 Posttranslational modifications 23

5 List of multimeric proteins 25

6 Complex lasso classification based on CATH database 26

7 Examples of proteins with various lasso structures 27

8 Analysis of proteins with small covalent loops 31

9 Mini-proteins 33

10 Structural alignment of proteins with L_6 lasso type 35

1 Construction of minimal surfaces

As described in the main manuscript, we define complex lassos as configurations in which backbone tails pierce through a surface spanned on loop formed by a part of the backbone chain. We note, that our procedure could be as well applied to any other well defined loop, possibly in other (bio)polymers. We classify lassos with respect to the number of crossings (piercings) through this surface. Note that on a fixed boundary (the covalent loop) in $\mathbb{R}^3$ one can span an infinite number of surfaces. Therefore an unambiguous definition and construction of such surface is crucial for our work.

In our analysis we have decided to work with minimal surfaces. Intuitively, mininmal surface is a surface that would be formed by a soap bubble spanned on a given

boundary. There are several equivalent definitions of such surfaces (connecting various mathematical disciplines). One of them is the local minimization condition: a surface $M \subset \mathbb{R}^3$ is minimal if and only if every point $p \in M$ has a neighborhood with the smallest area relative to its boundary. Notice that this is a local property: for a fixed global boundary there might be many such surfaces, possibly with different (smaller) global area. In our applications however surfaces determined with the local condition were sufficient. It can be shown that with appropriate assumptions the minimal surfaces can be equally well defined as a critical point of the Dirichlet energy functional, or as a surface with vanishing mean curvature.

In practical applications we need to work with discrete, triangulated versions of minimal surfaces, approximating the smooth surface. We construct such triangulated surfaces using discrete analogs of local area minimization and minimal Dirichlet energy conditions. The boundary of a triangulated surface is also discretized, being the polygonal chain in $\mathbb{R}^3$ with vertices in positions of $C\alpha$ atoms of the loop.

There are several algorithms, used in particular in computer graphics, that determine such triangulations. In our work we implemented a slightly modified version of an algorithm discussed in [1]. The initial data for this algorithm consists of coordinates of n vertices in the covalent loop, and the number of triangles in the triangulation that we are going to construct. This number allows to adjust the level of details of the resulting mesh – the larger the number, the surface is approximated more accurately. Once some initial mesh has been specified (as described in section 1.4 below), we iteratively adjust it by performing three operations that minimize the (local) area and the Dirichlet energy: Area Minimizing, Laplacian Fairing and Edge Swapping.

The scheme of applied algorithm is as follows [1]:

1. Initialization
2. Edge Swapping
3. DO {
4.   DO Laplacian Fairing WHILE (area change $> \epsilon_1$)
5.   Edge Swapping
6.   DO Area Minimizing WHILE (area change $> \epsilon_2$)
7.   Edge Swapping
8. } WHILE (area change $> \epsilon_0$)

Positive constants ϵ_0, ϵ_1 and ϵ_2 above are three fixed tolerance parameters – we quit the iteration if the modification of a triangulation in a given step does not change the surface area sufficiently (more than the relevant ϵ parameter).

In what follows we discuss each step of the algorithm in more detail. We use the similar notation as in [1]. A triangular mesh M is represented as a triple $\langle I, P, T \rangle$, where $I = \{1, 2, \ldots, N\}$ is the set of its vertices, $P\colon I \to \mathbb{R}^3$ is a mapping assigning each vertex

index its position in 3-dimensional space, and T is a set of triangles. Each triangle $t \in T$ is represented as an ordered triple $t = \langle i, j, k \rangle$ for $i, j, k \in I$ (with the vertices positions at $P(i), P(j)$ and $P(k)$). For simplicity we write $P_i \equiv P(i)$. Furthermore we assume that first n indices in I correspond to the fixed vertices of the boundary polygon (the covalent loop in a protein); to adjust the triangulation we can change locations of last $N - n$ vertices.

1.1 Area Minimizing

The area A of the mesh $M = \langle I, P, T \rangle$ is the sum of the areas of all triangles from the set T

$$A(M) = \sum_{t=\langle i,j,k \rangle \in T} \frac{1}{2} |P_j P_k \times P_j P_i|. \tag{1}$$

In the process of Area Minimizing (step 6 in our algorithm) we adjust coordinates $P_{n+1}, \ldots, P_N$ in order to minimize the value area functional $A(M)$. To this end we need to find a solution of the system of equations $\frac{\partial A(M)}{\partial P_h} = 0$, for all $h \in \{n+1, \ldots, N\}$. This set is equivalent to [1]:

$$\begin{aligned} P_h &= -\left(\sum_{\langle h,j,k \rangle \in NT(h)} \frac{(P_j P_k)^2 I_3 - (P_j P_k)(P_j P_k)^T}{|P_j P_k \times P_j P_h|} \right)^{-1} \\ &\quad \times \sum_{\langle h,j,k \rangle \in NT(h)} \frac{(P_j P_k \cdot P_j) P_j P_k - (P_j P_k)^2 P_j}{|P_j P_k \times P_j P_h|}, \end{aligned} \tag{2}$$

where $NT(i) \subseteq T$ is the set of all triangles containing vertex P_i, and I_3 is the 3×3 identity matrix. As the right-hand side of the above equation contains P_h, this is the iterative procedure of approximating the P_h position. This process can be repeated until the given tolerance ϵ_2 is reached.

1.2 Laplacian Fairing

With Laplacian Fairing we change the coordinates of mesh vertices in a way that minimizes the discrete version of Dirichlet energy functional. The continuous form of Dirichlet energy functional is

$$\frac{1}{2} \int_\Omega ||\nabla r(x)||^2 dV = \frac{1}{2} \int_\Omega \big(r_u^2(u,v) + r_v^2(u,v) \big) du dv, \tag{3}$$

where $r_u = \partial_u r$ and $r_v = \partial_v r$, Ω is a closed subset of $\mathbb{R}^2$ and $r \colon \Omega \to \mathbb{R}^3$ is a parametrization of the surface with boundary $\partial[r(\Omega)]$. It is known that critical points of the Dirichlet functional are harmonic functions, i.e. parametrizations r whose Laplacian vanishes, $\Delta r(u,v) = r_{uu} + r_{vv} = 0$. In discrete mesh we use a discrete version of the Laplacian, which at each vertex can be expressed by the so-called umbrella operator

$$\Delta(P_i) = \sum_{j \in N(i)} w_{ij} (P_j - P_i), \tag{4}$$

where $N(i) \subseteq I$ is the set of indices of vertices neighboring the vertex i, and w_{ij} are weights normalized so that $\sum_{j \in N(i)} w_{ij} = 1$ for each $i \in I$. As in the smooth case, the discrete Laplacian measures the difference between the value of a function at a particular point and the average of that function in its neighbors.

The weights w_{ij} in the formula (4) may be chosen in many different ways. In our implementation we define these weights by

$$w_{ij} = \frac{1}{2}(\mathrm{ctg}(\alpha_{1ij}) + \mathrm{ctg}(\alpha_{2ij})),$$

where α_{1ij} and α_{2ij} are angles in two triangles that share the edge P_iP_j, opposite to this edge. Our definition of weights differs slightly from the one given in [1]

In Laplacian Fairing we impose the condition that the discrete Laplacian vanishes, $\Delta(P_i) = 0$, for all interior vertices P_i, $i \in \{n+1, \ldots, N\}$. This leads to a system of $(N-n)$ (usually nonlinear) equations with $(N-n)$ variables, which cannot be solved explicitly, but enables to approximate each P_i iteratively with:

$$\overline{P_i} = \sum_{j \in N(i)} w_{ij} P_j, \tag{5}$$

where weights w_{ij} are computed from the old locations P_i and P_j and $\overline{P_i}$ is the new location.

1.3 Edge Swapping

In the process of Edge Swapping we change the connectivity of the mesh in the way that it minimizes the local area. We consider all pairs of triangles which share one edge (e.g. $t_1 = \langle i, j, h \rangle$ and $t_2 = \langle i, j, k \rangle$) and replace the common edge P_iP_j by the edge P_hP_k, if only it results in a triangulation of smaller area (see Fig. 1). Note that locations of the four vertices P_i, P_j, P_h, P_k remain unchanged – the only modification is in the connectivity of these vertices.

In a single Edge Swapping iteration we consider consecutively all edges in the mesh. If at least one swapping takes place (based on the above criteria) we continue this process. This operation is very fast and simple, however it returns just a local optimum (it is possible to find a global minimum, however with much higher computational cost).

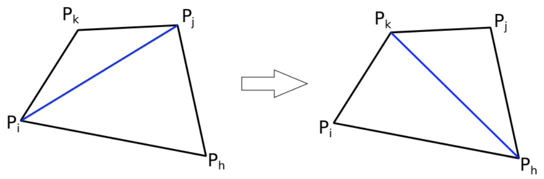

Figure 1: Edge Swapping.

1.4 Initialization

The initial mesh may be somehow arbitrary; we construct it as follows. First we specify the number of triangles m in the triangulation we are after. Second, we specify the polygonal boundary $P_1, \ldots, P_n$ consisting of (fixed) n points; to obtain more accurate triangulation, we can also divide boundary segments into shorter ones. Third, we compute the center of mass P_c of this boundary polygon and add s interior vertices along each line segment P_iP_c, $i \in \{1, 2, \ldots, n\}$. The number s is determined in order to obtain ca. m triangles in the mesh; some of these triangles are subsequently split into three smaller ones to get precisely m triangles in the mesh. Finally, we connect the vertices as shown in Fig. 2 (left panel). Our implementation differs slightly from original [1] shown in the right panel in Fig. 2. We reduce the number of edges sharing the center of mass P_c – this makes the central region of the triangulation less dense, and results in a smoother triangulated minimal surface.

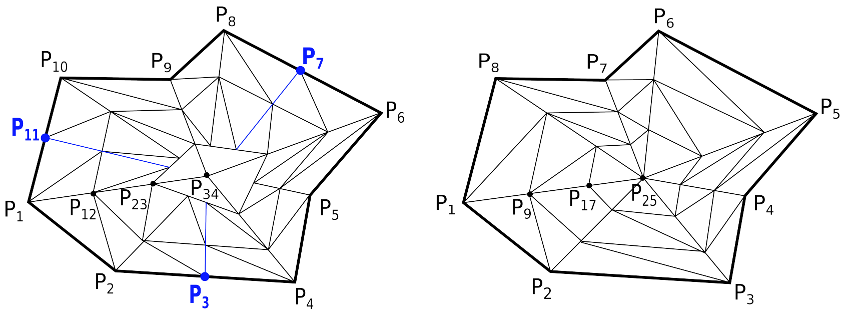

Figure 2: Left panel: an example of the initial mesh used in our algorithm, with three additional vertices P_3, P_7 and P_{11} in the boundary. Right panel: the initial mesh for the same input used in the implementation of the algorithm in [1].

1.5 Identification of lasso types

Once the triangulation of the minimal surface is determined we can verify which segments of the protein tail (or two tails) cross the surface. To identify a lasso type we also need to determine the direction of crossing (if only the surface is orientable which in our work always was the case). We denote the direction by drawing pierced triangles in different colors (e.g. in Fig. 3 blue and green triangles are pierced from opposite directions), and label the segments of a tail that pierce the surface with plus or minus signs respectively (e.g. tail segments denoted -10 and +289 in Fig. 3 pierce the surface from opposite directions).

Note that some proteins have complicated backbone configuration, giving rise to complicated, self-intersecting surfaces. In such cases it is convenient to present the triangulated surface as a planar barycentric embedding, in which each vertex of a triangulation is an average of vertices it is connected to. By a theorem by Tutte, such

representation can be uniquely determined purely from the connectivity structure of a triangular surface. We use a well known algorithm by Tutte [3] to determine such baricentric representation (with hyperbolic modification of the positions of the vertices in order to present it in a more pleasing way). As an example, such planar barycentric embedding for triangulated minimal surface spanned on a covalent loop in the protein with PDB code 3om0, is shown in Fig. 3.

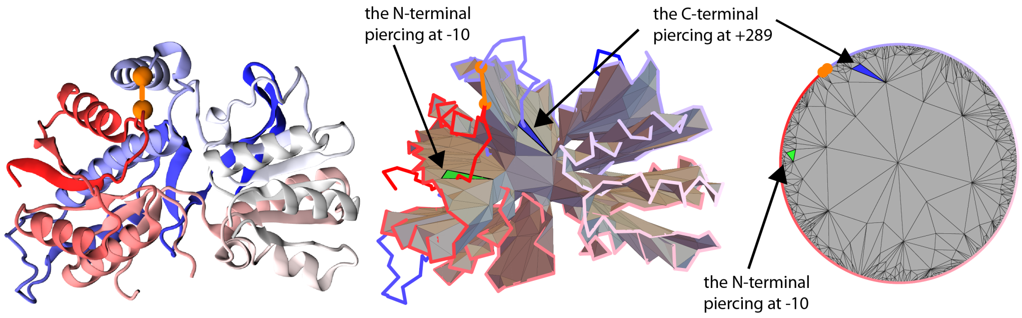

Figure 3: Left panel: cartoon representation of glutamate receptor, ionotropic kainate 5 protein (PDB code 3om0). Middle panel: triangulation of a minimal surface for 3om0 protein. The minimal surface, spanned on the covalent loop, is pierced twice by a tail (from opposite directions), through triangles in blue and green. Two cysteins and a cystein bond are shown in orange. Right panel: baricentric representation of a minimal triangulated surface for 3om0 protein. Two cysteins and a cystein bond comprise a part of the boundary and are shown in orange. Green and blue triangles are pierced from opposite sides by 10th and 289th tail segment respectively.

In our analysis we try not to include proteins whose lasso structure could be changed by thermal fluctuations. First, we impose a condition that there must be at least 10 amino acids separation between consecutive crossings (from opposite directions), i.e. a piece of a tail piercing a surface must be sufficiently "deep". There is one exception from this rule. Observe in Fig. 4 (right panel) that one may find a complex protein structure where a minimal surface spanned on a covalent loop, which has two distinct pieces located close to each other. In such case a tail may pierce both pieces of the surface and have less than 10 amino acids between these two crossings, but nonetheless we include such structures in our analysis. To detect such configurations automatically we compute (using Dijkstra algorithm) the shortest distance (along segments of the triangulation of the minimal surface) between two triangles that are pierced by a tail. If this distance is long enough (larger than 10 segments of the mesh) we include such a structure in our classification.

We also demand that the segment between the cysteine bridge and the first piercing includes at least 4 amino acids, see Fig. 4 (left panel).

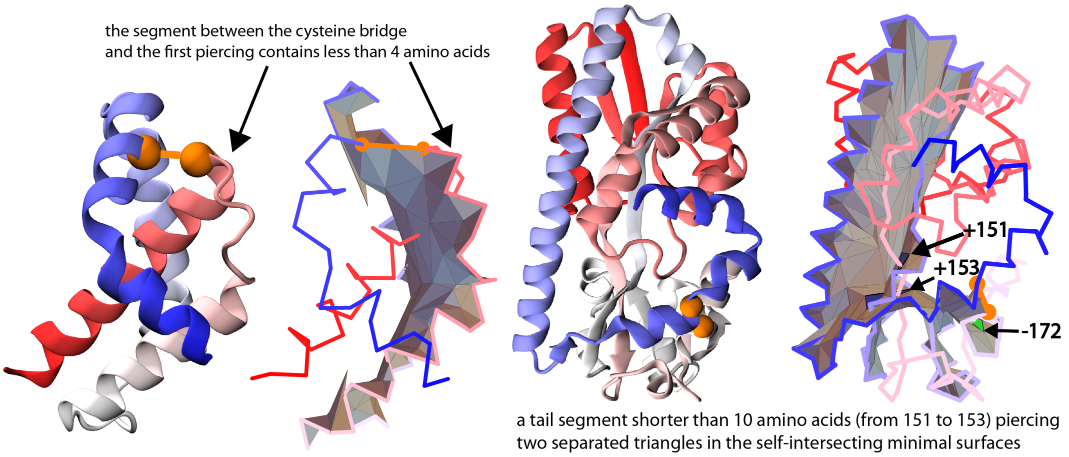

Figure 4: Left: in 3utk protein there are less than 4 amino acids between the cysteine bridge and the first piercing, therefore we don't include this protein in our analysis. Right: in 4p1e protein a short (less than 10 amino acids) tail segment pierces two separated triangles in a surface than is bent – included in our analysis.

2 Details of protein reconstruction

Homologue structures were identified with psi-blast algorithm (implemented in MODELLER software) run against all PDB sequences database provided by MODELLER team (http://salilab.org/modeller/downloads/pdball.pir.gz) with default parameters. Targets and template candidates were superimposed, and 3D alignment was calculated with Chimera [2] software. With these alignments, gaps coverage of found homologues was calculated. The first factor in the selection of a template was the overall percentage of the gap coverage in the alignment and the second the sequence similarity. All structures that had gaps longer than 8 amino acids without coverage were rejected. All structures left have been individually inspected with support of KnotProt database [4]. Doubtful structures were rejected.

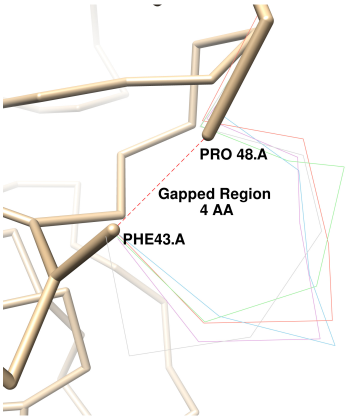

Figure 5: Example of a gapped structure (PDB code 1a7s chain A), with missing coordinates of 4 amino acids from the loop region, that were reconstructed with Modeller to determine a complex lasso type. Thick lines denote the original PDB structures (a backbone CA chain trace), thin lines mark modeled loops that were used to identify a lasso type.

Type	PDB Codes
A	1auk_A 1evs_A 1fsu_A 1g3p_A 1jvq_A 1lsh_A 1me8_A 1p49_A 1uhg_A 1z70_A 2bb3_A 2j04_B<br>2qqh_A 2wsd_A 2z04_A 2zf8_A 3a77_A 3c64_A 3ep1_A 3g89_A 3hi7_A 3kt7_A 3m03_A 3m8n_A<br>3nir_A 3nt1_A 3pgb_A 3sxx_A 3tw5_A 3vuo_A 3wky_A 4cvu_A 4d9i_A 4db5_A 4gqz_A 4kc3_B<br>4l0k_A 4l1d_A 4m7g_A 4mai_A 4ncd_A 4nn5_A 4o4y_L 4o5j_A 4o5p_B 4o65_A 4o6k_A 4oh3_A<br>4osn_A 4p79_A 4pmk_A 4r7q_A 4tmd_A 4uvq_A 4uxu_A 4wat_A
B	1a7s_A 1agq_A 1alu_A 1ax8_A 1b12_A 1b8k_A 1bcp_A 1bgc_A 1bqu_A 1bu8_A 1cru_A 1d2t_A<br>1dof_A 1dx4_A 1egi_A 1f0l_A 1f2q_A 1f32_A 1f97_A 1fcq_A 1fo8_A 1g5g_A 1gcy_A 1gku_B<br>1gml_A 1gv9_A 1h30_A 1hc1_A 1huw_A 1ijq_A 1jdp_A 1jnd_A 1js8_A 1jy5_A 1kl9_A 1kxo_A<br>1l1l_A 1lf7_A 1lml_A 1m48_A 1ms9_A 1n1f_A 1n8y_A 1ne2_A 1neu_A 1njr_A 1nko_A 1now_A<br>1nst_A 1o3u_A 1oi0_A 1olz_A 1omz_A 1p53_A 1p91_A 1pb7_A 1peq_A 1pew_A 1pgu_A 1pko_A<br>1ps1_A 1pwa_A 1pz7_A 1q35_A 1q8d_A 1qfo_A 1qfx_A 1qg8_A 1qgv_A 1qht_A 1r3e_A 1rxd_A<br>1s4n_A 1s5j_A 1scf_A 1so7_A 1sqj_A 1t6e_X 1tfz_A 1tzp_A 1u5x_A 1uct_A 1ups_A 1ux6_A<br>1uzk_A 1v0w_A 1v9m_A 1va6_A 1w07_A 1w8a_A 1w8k_A 1x9d_A 1xez_A 1xju_A 1yi9_A 1yis_A<br>1z4v_A 1ziw_A 1zk5_A 1zro_A 2aew_A 2arr_A 2b7u_A 2b9l_A 2bce_A 2bgh_A 2bog_X 2bou_A<br>2bsy_A 2c2a_A 2c9k_A 2cdc_A 2d1g_A 2d1h_A 2ddf_A 2ddu_A 2de0_A 2di4_A 2dre_A 2dvk_A<br>2e1v_A 2ecf_A 2eng_A 2fiy_A 2fj0_A 2fna_B 2fy7_A 2g5d_A 2gak_A 2gum_A 2gys_A 2h2t_A<br>2heh_A 2hft_A 2hlr_A 2hq4_A 2i10_A 2id5_A 2im9_A 2iy9_A 2j0a_A 2jd4_A 2jg0_A 2jju_A<br>2jks_A 2mpr_A 2nsm_A 2nw2_A 2nxf_A 2nyk_A 2o5n_A 2oay_A 2odp_A 2pf5_A 2pfc_A 2pmv_A<br>2pq6_A 2prs_A 2qki_A 2qn4_A 2r2j_A 2raa_A 2rag_A 2rl8_A 2uur_A 2uy2_A 2veq_A 2vl7_A<br>2vsm_A 2vxb_A 2w1z_A 2w2g_A 2w59_A 2w61_A 2w9x_A 2wjs_A 2wnf_A 2wnk_A 2wnv_A 2wy3_A<br>2x1q_A 2x2u_A 2x4i_A 2xlg_A 2xot_A 2xrc_A 2xu0_A 2y38_A 2y8d_A 2y8t_A 2yd6_A 2ydv_A<br>2yg2_A 2ykt_A 2ymo_A 2z2r_A 2z3q_A 2z4i_A 2zb6_A 2zou_A 2zws_A 2zxe_B 3agk_A 3ahq_A<br>3aja_A 3ajd_A 3ap1_A 3b1b_A 3b4n_A 3bix_A 3bqk_A 3bwu_D 3c3v_A 3ci0_A 3cj1_A 3cqn_A<br>3cwx_A 3czb_A 3d22_A 3db5_B 3dlq_I 3dxl_A 3e0g_A 3e2v_A 3ebw_A 3eeq_A 3erb_A 3f6k_A<br>3f95_A 3fgr_A 3fsa_A 3fvc_A 3fw3_A 3g4n_A 3g7n_A 3gax_A 3ghm_A 3gnz_A 3grf_A 3h09_A<br>3h2g_A 3h6g_A 3hhs_A 3hr6_A 3hsy_A 3i26_A 3i2t_A 3i6s_A 3i84_A 3icv_A 3ix0_A 3j0a_A<br>3jpw_A 3jxg_A 3k1l_A 3k1w_A 3k7b_A 3kbr_A 3kgl_A 3ks9_A 3ky9_A 3l4y_A 3llk_A 3lo8_A<br>3m19_A 3m1c_A 3m31_A 3m7p_A 3n7s_A 3nhi_A 3nk4_A 3nkq_A 3nsj_A 3nvx_A 3o22_A 3o6n_A<br>3odn_A 3oe3_A 3oen_A 3og6_A 3ojo_A 3okw_A 3om0_A 3omz_A 3p09_A 3pay_A 3pim_A 3pjz_A<br>3pow_A 3pv7_A 3pvk_A 3qcp_A 3qdh_A 3qek_A 3r1p_A 3rjo_A 3rm2_A 3rnq_A 3rty_A 3s26_A<br>3s98_A 3s9d_A 3sao_A 3sqr_A 3suu_A 3syj_A 3szh_A 3t0o_A 3t4l_A 3tc2_A 3thd_A 3tql_A<br>3u07_A 3u3l_A 3u74_U 3uan_A 3ub2_A 3ugf_A 3un7_A 3vpp_A 3vrh_A 3vta_A 3vu1_A 3vuu_A<br>3w2w_B 3w56_A 3weo_A 3whx_A 3wn4_A 3zgj_A 3zh5_A 3zib_A 3zxy_A 3zy2_A 3zyo_A 4a01_A<br>4adi_A 4ae2_A 4aee_A 4apm_A 4aqs_A 4art_A 4aru_A 4awe_A 4ay0_A 4b4h_A 4b87_A 4ba0_A<br>4bqy_A 4bsj_A 4buo_A 4bvn_A 4bwe_A 4c08_A 4cbp_A 4ccd_A 4cg1_A 4ci9_A 4cn9_A 4cu4_A<br>4cxp_A 4d8m_A 4d94_A 4dlo_A 4dlq_A 4dzr_A 4e6w_A 4eco_A 4ekx_A 4el6_A 4eme_A 4enz_A<br>4eyc_A 4fhq_A 4fr4_A 4ftb_A 4fww_A 4g2u_A 4gf2_A 4gqp_H 4h18_A 4hho_A 4hln_A 4hr9_A<br>4i0w_A 4i71_A 4iib_A 4ijy_A 4io2_A 4irm_A 4irp_A 4isc_A 4j2k_A 4j37_A 4j3r_A 4jd9_A<br>4jjh_A 4jjj_A 4job_A 4jqf_A 4jrn_A 4jvu_A 4jzz_A 4k3l_A 4k3y_A 4k60_A 4k8l_A 4kg7_A<br>4kgh_A 4kki_A 4kqa_A 4kt3_A 4kx7_A 4l7g_A 4le6_A 4lk4_A 4lv5_A 4lxr_A 4mh1_A 4mj2_A<br>4ms4_A 4msv_A 4myk_A 4myv_A 4mz2_A 4mz7_A 4nmx_A 4nob_A 4nqw_A 4nuu_A 4oe8_A 4p04_A<br>4p1e_A 4p49_A 4per_A 4plm_A 4rha_A 4tqg_A 4tr2_A 4v2d_A

Table 1: A. PDB codes of proteins with chain annotation (the last letter), with positions of some atoms along the chain not determined experimentally, and for which it was impossible to determine whether the repaired model has correct complex lasso type. B. PDB codes of proteins with chain annotation (the last letter), with positions of some atoms along the chain not determined experimentally, whose chains and complex lasso type were successfully modeled.

3 Full list of proteins

PDB code	Loop range	Function (classification)	Organism Species	Organism Genus
1AC5_A	79-345	Carboxypeptidase	Saccharomyces cerevisiae	Saccharomyces
1AHL_A	6-36	Neurotoxin	Anthopleura xanthogrammica	Anthopleura
1AHO_A	12-63	Neurotoxin	Androctonus australis	Androctonus
1AK0_A	72-217	Endonuclease	Penicillium citrinum	Penicillium
1AOC_A	60-161	Coagulation factor	Tachypleus tridentatus	Tachypleus
1AOZ_A	81-538	Oxidoreductase	Cucurbita pepo var. melopepo	Cucurbita
1ATA_A	22-60	Proteinase	Ascaris suum	Ascaris
1AX8_A	96-146	Cytokine	Homo sapiens	Homo
1B8W_A	16-32	Toxin	Ornithorhynchus anatinus	Ornithorhynchus
1BCP_A	41-201	Toxin	Bordetella pertussis	Bordetella
1BDS_A	6-32	Anti-hypertensive protein	Anemonia sulcata	Anemonia
1BEA_A	29-86	Serine protease inhibitor	Zea mays	Zea
1BF0_A	32-53	Calcium channel blocker	Dendroaspis angusticeps	Dendroaspis
1C01_A	11-64	Antimicrobial	Macadamia integrifolia	Macadamia
1C01_A	23-49	Antimicrobial	Macadamia integrifolia	Macadamia
1CCV_A	20-56	Hydrolase 3	Apis mellifera	Apis
1CFE_A	44-112	Pathogenesis-related	Solanum lycopersicum	Solanum
1CPY_A	56-298	Hydrolase	Saccharomyces cerevisiae	Saccharomyces
1CQ3_A	8-185	Cytokine	Cowpox virus	Orthopoxvirus
1D2S_A	164-188	Transport	Homo sapiens	Homo
1D6B_A	16-32	Toxin	Ornithorhynchus anatinus	Ornithorhynchus
1DOF_A	167-403	Lyase	Pyrobaculum aerophilum	Pyrobaculum
1DP4_A	164-213	Hormone/growth factor	Rattus norvegicus	Rattus
1DTV_A	18-62	Hydrolase	Hirudo medicinalis	Hirudo
1DTV_A	19-43	Hydrolase	Hirudo medicinalis	Hirudo
1DTV_A	22-58	Hydrolase	Hirudo medicinalis	Hirudo
1DYS_A	93-152	Cellulase	Humicola insolens	Humicola
1E4M_A	14-434	Hydrolase	Sinapis alba	Sinapis
1E4M_A	6-438	Hydrolase	Sinapis alba	Sinapis
1ESC_A	197-255	Hydrolase	Streptomyces scabiei	Streptomyces
1ETE_A	44-127	Cytokine	Homo sapiens	Homo
1FD3_A	15-30	Antimicrobial	Homo sapiens	Homo
1FJR_A	70-164	Signaling protein	Drosophila melanogaster	Drosophila
1FLC_A	126-174	Hydrolase	Influenza c virus	Influenzavirus C
1FLC_A	196-238	Hydrolase	Influenza c virus	Influenzavirus C
1FOB_A	253-311	Hydrolase	Aspergillus aculeatus	Aspergillus
1G66_A	147-179	Hydrolase	Penicillium purpurogenum	Talaromyces
1G6X_A	30-51	Hydrolase	Bos taurus	Bos
1GAK_A	60-134	Cell adhesion	Haliotis fulgens	Haliotis
1GP0_A	1598-1634	Receptor	Homo sapiens	Homo
1GXY_A	21-223	Transferase	Rattus norvegicus	Rattus
1H30_A	444-470	Growth arrest spec.	Homo sapiens	Homo
1H30_A	643-670	Growth arrest spec.	Homo sapiens	Homo
1HCN_B	23-72	Hormone	Homo sapiens	Homo
1HCN_B	26-110	Hormone	Homo sapiens	Homo
1HX2_A	21-60	Hydrolase	Bombina bombina	Bombina
1I1J_A	35-106	Hormone/growth factor	Homo sapiens	Homo

Continued on the next page

Table 2 – continued from the previous page

PDB code	Loop range	Function (classification)	Organism Species	Organism Genus
1I4U_A	51-173	Transport protein	Homarus gammarus	Homarus
1IJV_A	12-27	Defensin	Homo sapiens	Homo
1IYB_A	25-81	Hydrolase	Nicotiana glutinosa	Nicotiana
1JDP_A	168-216	Signaling protein	Homo sapiens homo sapiens	Homo
1JER_A	60-95	Electron transport	Cucumis sativus	Cucumis
1JFU_B	10-155	Membrane	Bradyrhizobium japonicum	Bradyrhizobium
1JLI_A	16-84	Cytokine	Homo sapiens	Homo
1JY5_A	26-84	Hydrolase	Calystegia sepium	Calystegia
1JY5_A	57-90	Hydrolase	Calystegia sepium	Calystegia
1KJ6_A	18-33	Antibiotic	Homo sapiens	Homo
1KTH_A	30-51	Structural protein	Homo sapiens	Homo
1KXO_A	42-170	Ligand binding	Pieris brassicae	Pieris
1LE6_A	48-122	Hydrolase	Homo sapiens	Homo
1LKI_A	12-134	Cytokine	Mus musculus	Mus
1LKI_A	18-131	Cytokine	Mus musculus	Mus
1M4L_A	138-161	Hydrolase	Bos taurus	Bos
1MC2_A	1050-1134	Toxin	Deinagkistrodon acutus	Deinagkistrodon
1MEG_A	153-204	Hydrolase	Carica papaya	Carica
1MJN_A	161-299	Immune system	Homo sapiens	Homo
1N1F_A	10-103	Immune system	Homo sapiens	Homo
1N2Z_A	183-259	Transport protein	Escherichia coli	Escherichia
1NF2_A	35-265	Structural protein	Thermotoga maritima	Thermotoga
1NSC_A	86-419	Hydrolase(o-glycosyl)	Influenza B virus	Influenzavirus B
1NYO_A	8-142	Immune system	Mycobacterium tuberculosis	Mycobacterium
1OH1_A	16-55	Cysteine proteinase inh.	Staphylococcus aureus	Staphylococcus
1OK0_A	45-73	Inhibitor	Streptomyces tendae	Streptomyces
1PB7_A	236-290	Ligand binding	Rattus norvegicus	Rattus
1PZ7_A	175-201	Structural protein	Gallus gallus	Gallus
1PZS_A	54-165	Oxidoreductase	Mycobacterium tuberculosis	Mycobacterium
1Q25_A	385-419	Protein binding	Bos taurus	Bos
1Q25_A	81-111	Protein binding	Bos taurus	Bos
1Q77_A	14-114	Structural protein	Aquifex aeolicus	Aquifex
1QCX_A	72-206	Lyase	Aspergillus niger	Aspergillus
1QFT_A	48-169	Ligand binding	Rhipicephalus appendiculatus	Rhipicephalus
1QG8_A	155-243	Transferase	Bacillus subtilis	Bacillus
1QGV_A	38-79	Transcription	Homo sapiens	Homo
1R8N_A	44-89	Hydrolase	Delonix regia	Delonix
1SCF_A	43-138	Hormone/growth factor	Homo sapiens	Homo
1SGL_A	26-84	Hydrolase	Trichosanthes lepiniana	Trichosanthes
1SGL_A	57-90	Hydrolase	Trichosanthes lepiniana	Trichosanthes
1SHI_A	5-33	Neurotoxin	Stichodactyla helianthus	Stichodactyla
1SVB_A	74-105	Viral	Tick-borne encephalitis virus	Flavivirus
1T61_A	164-222	Structural protein	Bos taurus	Bos
1T61_A	53-108	Structural protein	Bos taurus	Bos
1TAP_A	33-55	Proteinase	Ornithodoros moubata	Ornithodoros
1TZP_A	44-265	Hydrolase	Escherichia coli	Escherichia
1U53_A	65-148	Antibiotic	Necator americanus	Necator
1UDK_A	20-41	Unknown	Naja nigricollis	Naja
1UDK_A	7-37	Unknown	Naja nigricollis	Naja

Continued on the next page

Table 2 – continued from the previous page

PDB code	Loop range	Function (classification)	Organism Species	Organism Genus
1UWC_A	29-258	Hydrolase	Aspergillus niger	Aspergillus
1UZK_A	1549-1574	Glycoprotein	Homo sapiens	Homo
1VF8_A	28-373	Immune system	Mus musculus	Mus
1W8K_A	265-363	Antigen	Plasmodium vivax	Plasmodium
1W8K_A	388-444	Antigen	Plasmodium vivax	Plasmodium
1WC2_A	30-69	Hydrolase	Mytilus edulis	Mytilus
1WC2_A	65-178	Hydrolase	Mytilus edulis	Mytilus
1WC2_A	72-157	Hydrolase	Mytilus edulis	Mytilus
1WKT_A	11-72	Toxin	Williopsis saturnus var. mrakii	Cyberlindnera
1WKT_A	27-58	Toxin	Williopsis saturnus var. mrakii	Cyberlindnera
1WQK_A	6-30	Toxin	Anthopleura elegantissima	Anthopleura
1WS8_A	58-92	Electron transport	Cucurbita pepo	Cucurbita
1X8Q_A	41-171	Ligand binding	Rhodnius prolixus	Rhodnius
1XTA_A	56-134	Toxin	Naja atra	Naja
1XTM_B	93-186	Structural protein	Bacillus subtilis	Bacillus
1YG9_A	51A-113	Hydrolase	Blattella germanica	Blattella
1YI9_A	81-126	Oxidoreductase	Rattus norvegicus	Rattus
1YS1_A	190-270	Hydrolase	Burkholderia cepacia	Burkholderia
1ZMI_A	3-18	Antimicrobial	Homo sapiens	Homo
1ZMM_A	4-19	Antimicrobial	Homo sapiens	Homo
2B7U_A	217-254	Hydrolase	Charybdis maritima	Drimia
2B9L_A	69-105	Immune system	Holotrichia diomphalia	Holotrichia
2BB6_A	3-252	Transport protein	Bos taurus	Bos
2BGH_A	25-89	Transferase	Rauvolfia serpentina	Rauvolfia
2C1C_A	138-161	Hydrolase	Helicoverpa zea	Helicoverpa
2CKS_A	166-406	Hydrolase	Thermobifida fusca	Thermobifida
2CMZ_A	68-114	Membrane	Vesicular stomatitis indiana virus	Vesiculovirus
2D5W_A	314-458	Peptide binding	Thermus thermophilus	Thermus
2DDU_A	1475-1522	Signaling	Mus musculus	Mus
2DRE_A	45-92	Plant protein	Lepidium virginicum	Lepidium
2E1V_A	125-433	Transferase	Chrysanthemum x morifolium	Chrysanthemum
2ENG_A	16-86	Hydrolase	Humicola insolens	Humicola
2ENG_A	87-199	Hydrolase	Humicola insolens	Humicola
2ENG_A	89-189	Hydrolase	Humicola insolens	Humicola
2ERF_A	153-214	Sugar	Homo sapiens	Homo
2F5X_A	142-178	Transport protein	Bordetella pertussis tohama I	Bordetella
2FMA_A	144-174	Metal binding	Homo sapiens	Homo
2G5X_A	32-214	Hydrolase	Lychnis chalcedonica	Silene
2GHV_E	366-419	Viral	Sars coronavirus	Betacoronavirus
2GUM_A	364-412	Viral protein	Human herpesvirus 1	Simplexvirus
2HCZ_X	42-70	Allergen 2	Zea mays	Zea
2HCZ_X	73-140	Allergen 2	Zea mays	Zea
2IKD_A	23-54	Hydrolase	Manduca sexta	Manduca
2IKE_A	83-113	Hydrolase	Manduca sexta	Manduca
2J6D_A	35-56	Toxin	Conus striatus	Conus
2JD4_A	2845-2870	Metal binding	Mus musculus	Mus
2JD4_A	3024-3055	Metal binding	Mus musculus	Mus
2JIG_A	195-230	Hydrolase	Chlamydomonas reinhardtii	Chlamydomonas
2JKS_A	66-77	Immune system	Toxoplasma gondii	Toxoplasma

Continued on the next page

Table 2 – continued from the previous page

PDB code	Loop range	Function (classification)	Organism Species	Organism Genus
2JON_A	48-94	Allergen	Olea europaea	Olea
2JOP_A	60-125	Immune system	Homo sapiens	Homo
2JR3_A	16-32	Antimicrobial	Pelodiscus sinensis	Pelodiscus
2JTO_A	10-27	Hydrolase	Rhipicephalus bursa	Rhipicephalus
2JTO_A	47-64	Hydrolase	Rhipicephalus bursa	Rhipicephalus
2JX9_A	41-71	Cell adhesion	Mus musculus	Mus
2K8P_A	70-124	Signaling	Homo sapiens	Homo
2KER_A	43-70	Hydrolase	Streptomyces parvulus	Streptomyces
2KQA_A	20-57	Toxin	Ceratocystis platani	Ceratocystis
2KQA_A	60-115	Toxin	Ceratocystis platani	Ceratocystis
2KXI_A	67-128	Transferase	Neisseria meningitidis serogroup b	Neisseria
2L3O_A	43-106	Cytokine	Mus musculus	Mus
2LVX_A	408-437	Hydrolase	Schizosaccharomyces pombe	Schizosaccharomyces
2MJK_A	12-28	Antimicrobial	Gallus gallus	Gallus
2MM2_A	18-65	Plant protein	Pyrenophora tritici-repentis	Pyrenophora
2MN3_A	16-30	Antimicrobial	Ornithorhynchus anatinus	Ornithorhynchus
2OIZ_D	130-161	Oxidoreductase	Alcaligenes faecalis	Alcaligenes
2OR7_A	38-90	Immune system	Mus musculus	Mus
2OYA_A	446-507	Ligand	Mus musculus	Mus
2PE4_A	43-333	Hydrolase	Homo sapiens	Homo
2PMV_A	8-228	Transport protein	Homo sapiens	Homo
2PSP_A	58-84	Signaling	Sus scrofa	Sus
2PSP_A	8-35	Signaling	Sus scrofa	Sus
2PT5_A	10-110	Transferase	Aquifex aeolicus	Aquifex
2Q9O_A	298-332	Oxidoreductase	Melanocarpus albomyces	Melanocarpus
2QN4_A	41-90	Hydrolase	Oryza sativa subsp. japonica	Oryza
2QRL_A	205-249	Oxidoreductase	Saccharomyces cerevisiae	Saccharomyces
2RL8_A	106-141	Protein transport	Bos taurus	Bos
2RNG_A	52-70	Antimicrobial	Tachypleus tridentatus	Tachypleus
2UUR_A	175-229	Structural protein	Homo sapiens	Homo
2UUX_A	24-51	Inhibitor	Rhipicephalus appendiculatus	Rhipicephalus
2UUX_A	52-69	Inhibitor	Rhipicephalus appendiculatus	Rhipicephalus
2VEC_A	10-204	Cytosolic	Escherichia coli	Escherichia
2VGA_A	33-199	Viral	Vaccinia virus	Orthopoxvirus
2W2G_A	492-623	Rna-binding	Sars coronavirus	Betacoronavirus
2W61_A	390-442	Glycoprotein	Saccharomyces cerevisiae	Saccharomyces
2W8X_A	51-69	Membrane	Rhipicephalus appendiculatus	Rhipicephalus
2W9X_A	165-333	Hydrolase	Cellvibrio japonicus	Cellvibrio
2WB9_A	26-196	Transferase	Fasciola hepatica	Fasciola
2WBF_X	755-809	Hydrolase	Plasmodium falciparum	Plasmodium
2WNK_A	179-218	Membrane protein	Toxoplasma gondii	Toxoplasma
2X46_A	50-155	Allergen	Argas reflexus	Argas
2XFD_A	90-101	Sugar	Escherichia coli	Escherichia
2XRC_A	123-163	Immune system	Homo sapiens	Homo
2XU3_A	156-209	Hydrolase	Homo sapiens	Homo
2Y1B_A	74-118	Membrane	Escherichia coli	Escherichia
2Y8T_A	304-393	Membrane	Toxoplasma gondii	Toxoplasma
2YDV_A	71-159	Receptor	Homo sapiens	Homo
2Z4I_A	145-211	Signaling	Escherichia coli	Escherichia

Continued on the next page

Table 2 – continued from the previous page

PDB code	Loop range	Function (classification)	Organism Species	Organism Genus
2ZK9_A	76-172	Hydrolase	Chryseobacterium proteolyticum	Chryseobacterium
2ZK9_A	77-126	Hydrolase	Chryseobacterium proteolyticum	Chryseobacterium
2ZWS_A	322-370	Hydrolase	Pseudomonas aeruginosa	Pseudomonas
2ZX2_A	106-135	Immune system	Oncorhynchus keta	Oncorhynchus
2ZX2_A	6-35	Immune system	Oncorhynchus keta	Oncorhynchus
3A2E_A	10-86	Plant protein	Ginkgo biloba	Ginkgo
3AIH_A	181-216	Sugar binding	Homo sapiens	Homo
3BRN_A	40-153	Ligand binding	Argas monolakensis	Argas
3BWK_A	177-238	Hydrolase	Plasmodium falciparum	Plasmodium
3CQN_A	118-249	Oxidoreductase	Arabidopsis thaliana	Arabidopsis
3CTK_A	32-212	Hydrolase	Bougainvillea spectabilis	Bougainvillea
3D22_A	4-58	Oxidoreductase	Populus trichocarpa	Populus
3DB5_B	49-124	Transferase	Homo sapiens	Homo
3DJL_A	28-540	Oxidoreductase	Escherichia coli	Escherichia
3DUZ_A	128-158	Viral protein	Autographa cal. nuc. pol. virus	Alphabaculovirus
3EBW_A	41-162	Allergen	Periplaneta americana	Periplaneta
3EDH_A	42-198	Hydrolase	Homo sapiens	Homo
3EDY_A	365-526	Hydrolase	Homo sapiens	Homo
3EQN_A	5-424	Hydrolase	Phanerochaete chrysosporium	Phanerochaete
3F5V_A	4-117	Hydrolase	Dermatophagoides pteronyssinus	Dermatophagoides
3FLP_A	184-215	Sugar binding	Limulus polyphemus	Limulus
3G7N_A	25-254	Hydrolase	Penicillium expansum	Penicillium
3HEI_B	80-140	Transferase	Homo sapiens	Homo
3I26_A	108-156	Hydrolase	Breda virus serotype 1	Torovirus
3I5W_A	5-20	Antimicrobial	Homo sapiens	Homo
3JXG_A	78-261	Cell adhesion	Mus musculus	Mus
3L49_A	166-224	Transport protein	Rhodobacter sphaeroides	Rhodobacter
3L91_A	67-148	Hydrolase	Pseudomonas aeruginosa	Pseudomonas
3LQB_A	50-199	Hydrolase	Danio rerio	Danio
3M31_A	90-349	Oxidoreductase	Saccharomyces cerevisiae	Saccharomyces
3MB5_A	196-233	Transferase	Pyrococcus abyssi	Pyrococcus
3MTW_A	172-213	Hydrolase	Caulobacter vibrioides	Caulobacter
3NGG_A	10-35	Antibiotic	Oxyuranus microlepidotus	Oxyuranus
3NGW_A	24-192	Biosynthetic protein	Archaeoglobus fulgidus	Archaeoglobus
3NK4_A	251-335	Cell adhesion	Gallus gallus	Gallus
3NKQ_A	148-194	Hydrolase	Mus musculus	Mus
3OEN_A	229-284	Transport protein	Rattus norvegicus	Rattus
3ON9_A	180-317	Viral	Ectromelia virus	Orthopoxvirus
3OP8_A	288-326	Protein	Homo sapiens	Homo
3OZP_A	36-55	Hydrolase	Ostrinia furnacalis	Ostrinia
3PIV_A	4-99	Cytokine	Danio rerio	Danio
3PIW_A	6-101	Cytokine	Danio rerio	Danio
3Q2U_A	75-156	Membrane	Homo sapiens	Homo
3Q31_A	58-219	Lyase	Aspergillus oryzae	Aspergillus
3QDH_A	394-445	Cell adhesion	Actinomyces naeslundii	Actinomyces
3QSD_A	133-199	Hydrolase	Schistosoma mansoni	Schistosoma
3QTE_A	6-20	Antimicrobial	Homo sapiens	Homo
3QVP_A	164-206	Oxidoreductase	Aspergillus niger	Aspergillus
3QW9_A	84-153	Cytokine	Rattus norvegicus	Rattus

Continued on the next page

Table 2 – continued from the previous page

PDB code	Loop range	Function (classification)	Organism Species	Organism Genus
3RLG_A	53-201	Hydrolase	Loxosceles intermedia	Loxosceles
3S8K_A	45-89	Hydrolase	Carica papaya	Carica
3SH4_A	159-193	Metal binding	Homo sapiens	Homo
3SUK_A	39-76	Unknown	Moniliophthora perniciosa	Moniliophthora
3SUK_A	79-138	Unknown	Moniliophthora perniciosa	Moniliophthora
3SUM_A	43-80	Unknown	Moniliophthora perniciosa	Moniliophthora
3SUM_A	83-145	Unknown	Moniliophthora perniciosa	Moniliophthora
3T0O_A	202-213	Hydrolase	Homo sapiens	Homo
3T94_A	138-205	Transferase	Sulfolobus solfataricus	Sulfolobus
3TC2_A	48-97	Hydrolase	Solanum tuberosum	Solanum
3U4Y_A	48-319	Structural protein	Desulfotomaculum acetoxidans	Desulfotomaculum
3U74_A	115-147	Hydrolase receptor	Homo sapiens	Homo
3U74_A	95-122	Hydrolase receptor	Homo sapiens	Homo
3UTK_N	61-115	Protein	Dickeya dadantii	Dickeya
3UYX_N	96-142	Viral	Influenza A virus	Influenza A
3V5A_N	425-647	Metal binding protein	Bos taurus	Bos
3V83_C	402-674	Metal binding protein	Homo sapiens	Homo
3V83_C	418-637	Metal binding protein	Homo sapiens	Homo
3VUP_A	177-244	Hydrolase	Aplysia kurodai	Aplysia
3VX0_A	440-475	Hydrolase	Aspergillus oryzae	Aspergillus
3WMT_A	76-129	Hydrolase	Aspergillus oryzae	Aspergillus
3WP4_A	4-172	Hydrolase	Neocallimastix patriciarum	Neocallimastix
3ZC9_A	41-85	Hydrolase	Murraya koenigii	Murraya
3ZPX_A	101-273	Hydrolase	Ustilago maydis	Ustilago
3ZUI_A	56-168	Immune system	Ornithodoros moubata	Ornithodoros
3ZXC_A	3-26	Signaling	Cupiennius salei	Cupiennius
3ZY2_A	266-353	Transferase	Caenorhabditis elegans	Caenorhabditis
4A56_A	53-107	Protein binding	Klebsiella oxytoca	Klebsiella
4A7U_A	57-146	Oxidoreductase	Homo sapiens	Homo
4ADI_A	49-287	Viral	Rubella virus	Rubivirus
4ADI_A	51-130	Viral	Rubella virus	Rubivirus
4B7Q_C	92-417	Hydrolase	Influenza a virus	Influenza A
4BOE_A	28-150	Cholesterol binding	Rhipicephalus appendiculatus	Rhipicephalus
4BQD_A	51-72	Blood clotting	Homo sapiens	Homo
4CMR_A	193-303	Hydrolase	Pyrococcus sp. st04	Pyrococcus
4CXP_A	66-218	Hydrolase	Arabidopsis thaliana	Arabidopsis
4CYL_A	111-309	Cell adhesion	Caenorhabditis elegans	Caenorhabditis
4CYL_A	113-155	Cell adhesion	Caenorhabditis elegans	Caenorhabditis
4D8M_A	414-489	Lipid	Bacillus thuringiensis	Bacillus
4ETR_A	30-101	Unknown	Pseudomonas aeruginosa	Pseudomonas
4F23_A	95-138	Viral	Influenza A virus	Influenza A
4FDI_A	308-419	Hydrolase	Homo sapiens	Homo
4FNK_C	97-139	Viral	Influenza A virus	Influenza A
4G2U_A	74-152	Immune system	Ostertagia ostertagi	Ostertagia
4GDI_A	92-417	Viral	Influenza A virus	Influenza A
4GE1_A	42-176	Amine-binding	Rhodnius prolixus	Rhodnius
4GQR_A	70-115	Hydrolase	Homo sapiens	Homo
4GV5_A	11-30	Toxin	Crotalus durissus terrificus	Crotalus
4GWN_N	103-255	Hydrolase	Homo sapiens	Homo

Continued on the next page

Table 2 – continued from the previous page

PDB code	Loop range	Function (classification)	Organism Species	Organism Genus
4H14_A	21-165	Viral	Bovine coronavirus	Betacoronavirus
4HJ1_A	756-852	Viral	Rift valley fever virus	Phlebovirus
4HJ1_A	777-825	Viral	Rift valley fever virus	Phlebovirus
4HLN_A	126-506	Transferase	Hordeum vulgare	Hordeum
4HS9_A	181-238	Hydrolase	Proteus mirabilis	Proteus
4HYQ_A	152-199	Hydrolase	Streptomyces albidoflavus	Streptomyces
4I71_A	199-304	Hydrolase	Trypanosoma brucei brucei	Trypanosoma
4IGT_A	739-794	Membrane protein	Rattus norvegicus	Rattus
4IHZ_A	33-80	Hydrolase	Crataeva tapia	Crateva
4IO2_A	193-247	Membrane	Adineta vaga	Adineta
4J37_A	142-196	Rna binding protein	Homo sapiens	Homo
4JD0_A	32-241	Transferase	Thermotoga maritima	Thermotoga
4JJO_A	23-48	Sugar binding	Clavibacter michiganensis	Clavibacter
4JP6_A	29-61	Unknown	Carica papaya	Carica
4JP6_A	64-120	Unknown	Carica papaya	Carica
4JPH_A	87-137	Cytokine	Mus musculus	Mus
4JWO_A	154-263	Phosphate binding	Planctomyces limnophilus	Planctopirus
4KK7_A	150-345	Protein binding	Mycobacterium tuberculosis	Mycobacterium
4KKI_A	3-242	Transport protein	Homo sapiens	Homo
4KNC_A	44-229	Sugar binding	Pseudomonas aeruginosa	Pseudomonas aeruginosa
4KP1_A	102-365	Isomerase	Methanocaldococcus jannaschii	Methanocaldococcus
4KYP_A	43-69	Toxin	Hottentotta judaicus	Hottentotta
4L05_A	55-150	Oxidoreductase	Brucella abortus	Brucella
4L3N_A	425-478	Viral	Human betacoronavirus 2c	Betacoronavirus
4L7G_A	217-382	Hydrolase	Homo sapiens	Homo
4LB1_A	4-19	Antimicrobial	Homo sapiens	Homo
4LBF_A	4-19	Antimicrobial protein	Homo sapiens	Homo
4LQ6_A	33-81	Hydrolase	Mycobacterium tuberculosis	Mycobacterium
4MYK_A	35-242	Hydrolase	Trypanosoma cruzi	Trypanosoma
4N03_A	89-347	Transport protein	Thermomonospora curvata	Thermomonospora
4N3T_A	87-162	Oxidoreductase	Candida albicans	Candida
4N7C_A	44-175	Protein binding	Blattella germanica	Blattella
4NT5_A	2739-2788	Protein binding	Homo sapiens	Homo
4OIE_A	280-329	Viral	West nile virus	Flavivirus
4OIE_A	291-312	Viral	West nile virus	Flavivirus
4P02_B	163-430	Transferase	Rhodobacter sphaeroides	
4P27_A	56-130	Allergen	Schistosoma mansoni	Schistosoma
4PLM_A	121-154	Protein binding	Gallus gallus	Gallus
4R2B_A	290-357	Transport	Ochrobactrum anthropi	Ochrobactrum
4TLP_A	44-88	Hydrolase	Psophocarpus tetragonolobus	Psophocarpus

Table 2: Protein chains with a single lasso (L_1 type), i.e. with loops pierced once by a tail. **In total 331 loops pierced once have been identified in 296 proteins.**

PDB code	Loop range	Function (classification)	Organism Species	Organism Genus
1AOC_A	10-95	Coagulation factor	Tachypleus tridentatus	Tachypleus
1BR9_A	1-72	Proteinase	Homo sapiens	Homo
1ETE_A	93-132	Cytokine	Homo sapiens	Homo
1F2L_A	8-34	Cytokine	Homo sapiens	Homo
1G0Y_R	104-147	Immune system	Homo sapiens	Homo
1GVZ_A	22-157	Hydrolase	Equus caballus	Equus
1HC1_A	562-609	Oxygen transport	Panulirus interruptus	Panulirus
1KKH_A	112-286	Transferase	Methanocaldococcus jannaschii	Methanocaldococcus
1M8A_A	6-32	Cytokine	Homo sapiens	Homo
1NR4_A	10-34	Cytokine	Homo sapiens	Homo
1O7Z_A	9-36	Chemokine	Homo sapiens	Homo
1OMZ_A	244-296	Transferase	Mus musculus	Mus
1QFX_A	109-453	Hydrolase	Aspergillus niger	Aspergillus
1RJT_A	9-36	Cytokine	Homo sapiens	Homo
1TVX_A	25-51	Cytokine	Homo sapiens	Homo
1XWE_A	1514-1588	Signaling	Homo sapiens	Homo
1YPY_A	49-136	Viral protein	Vaccinia virus	Orthopoxvirus
1ZPU_A	102-521	Oxidoreductase	Saccharomyces cerevisiae	Saccharomyces
1ZXT_A	12-36	Signaling	Human herpesvirus 8	Rhadinovirus
2FFU_A	345-423	Transferase	Homo sapiens	Homo
2GMF_A	88-121	Growth factor	Homo sapiens	Homo
2HDL_A	3-29	Cytokine	Homo sapiens	Homo
2LT5_C	3-78	Hydrolase	Rana pipiens	Rana
2OIZ_D	81-113	Oxidoreductase	Alcaligenes faecalis	Alcaligenes
2P3X_A	25-88	Oxidoreductase	Vitis vinifera	Vitis
2Q9O_A	114-540	Oxidoreductase	Melanocarpus albomyces	Melanocarpus
2RA4_A	11-35	Cytokine	Homo sapiens	Homo
2VGA_A	6-166	Viral	Vaccinia virus	Orthopoxvirus
2X97_C	467-612	Hydrolase	Drosophila melanogaster	Drosophila
2YAU_A	89-213	Oxidoreductase	Leishmania infantum	Leishmania
3F95_A	768-811	Hydrolase	Pseudoalteromonas sp.	Pseudoalteromonas
3GV3_A	9-34	Cytokine	Homo sapiens	Homo
3HHS_A	586-630	Oxidoreductase	Manduca sexta	Manduca
3HHS_A	588-637	Oxidoreductase	Manduca sexta	Manduca
3NKQ_A	156-350	Hydrolase	Mus musculus	Mus
3NSW_C	3-62	Immune system	Ancylostoma ceylanicum	Ancylostoma
3PXL_A	85-488	Oxidoreductase	Trametes hirsuta	Trametes
3RT4_A	22-67	Immune system	Camelus dromedarius	Camelus
3SQR_A	108-524	Oxidoreductase	Botrytis aclada	Botrytis
3TM0_A	19-156	Transferase/antibiotic	Enterococcus faecalis	Enterococcus
3TN2_A	11-35	Cytokine	Homo sapiens	Homo
3ZK4_A	203-367	Oxidoreductase	Lupinus luteus	Lupinus
4ADI_A	37-242	Viral protein	Rubella virus	Rubivirus
4HCS_A	15-40	Signaling	Danio rerio	Danio
4HWM_A	68-124	Unknown	Klebsiella pneumoniae	Klebsiella
4N1I_A	10-278	Hydrolase	Ustilago maydis	Ustilago
4PSC_A	55-91	Hydrolase	Trichoderma reesei	Trichoderma

Table 3: Protein chains with a double lasso (L_2 type), i.e. with loops pierced twice by a tail. **In total 47 loops pierced twice have been identified in 46 proteins.**

**PDB code**	**Loop range**	**Function (classification)**	**Organism Species**	**Organism Genus**
1BJ7_A	63-154	Allergen	Bos taurus	Bos
1DZK_A	63-155	Transport	Sus scrofa	Sus
1EPA_A	60-154	Retinoic	Rattus norvegicus	Rattus
1KT6_A	70-174	Transport	Bos taurus	Bos
1LF7_A	76-168	Immune	Homo sapiens	Homo
1U3D_A	80-190	Signaling	Arabidopsis thaliana	Arabidopsis
2EHG_A	58-145	Hydrolase	Sulfolobus tokodaii	Sulfolobus
2L5P_A	98-203	Transport	Rattus norvegicus	Rattus
2RA6_A	73-166	Transport	Trichosurus vulpecula	Trichosurus
2VGA_A	112-152	Viral	Vaccinia virus	Orthopoxvirus
2YG2_A	95-183	Lipid transport	Homo sapiens	Homo
3AGN_A	1-54	Hydrolase	Ustilago sphaerogena	Ustilago
3EEQ_A	60-285	Structural	Sulfolobus solfataricus	Sulfolobus
3FIQ_A	63-155	Transport	Rattus norvegicus	Rattus
3KFF_A	64-157	Transport	Mus musculus	Mus
3KQ0_A	72-165	Signaling	Homo sapiens	Homo
3L4R_A	64-157	Allergen,	Canis familiaris	Canis
3NSJ_A	241-407	Immune	Mus musculus	Mus
3O22_A	89-186	Isomerase	Homo sapiens	Homo
3QL6_A	6-167	Oxidoreductase	Bos taurus	Bos
3S26_A	78-177	Transport	Mus musculus	Mus
3SAO_A	58-151	Transport	Gallus gallus	Gallus
4CK4_A	66-160	Transport	Ovis aries	Ovis
4H14_A	172-252	Viral	Bovine coronavirus	Betacoronavirus
4ODD_B	62-154	Allergen	Canis lupus familiaris	Canis

Table 4: Protein chains with a triple lasso (L_3 type), i.e. with loops pierced three times by a tail. **In total 25 loops triply pierced have been identified in 25 proteins.**

**PDB code**	**Loop range**	**Function (classification)**	**Organism Species**	**Organism Genus**
4QI7_A	167-211	Oxidoreductase	Neurospora crassa	Neurospora

Table 5: Protein chains with a sixfold lasso (L_6 type), i.e. with loops pierced six times by a tail. **In total 1 loop pierced six times has been identified in 1 protein.**

PDB code	Loop range	Function (classification)	Organism Species	Organism Genus	Type
2D1G_A	216-269	Hydrolase	Francisella tularensis subsp. novicida	Francisella	$L_{1,1}$
2DVZ_A	93-152	Transport protein	Bordetella pertussis	Bordetella	
2YHG_A	564-779	Hydrolase	Saccharophagus degradans	Saccharophagus	
3OM0_A	17-273	Membrane	Rattus norvegicus	Rattus	
3WA1_A	67-161	Toxin	Lysinibacillus sphaericus	Lysinibacillus	
4A3X_C	78-119	Cell adhesion	Candida glabrata	Nakaseomyces	
4ASL_A	78-119	Cell adhesion	Candida glabrata	Nakaseomyces	
2CMZ_A	177-224	Membrane protein	Vesicular stomatitis indiana virus	Vesiculovirus	
4JGL_A	57-142	Structural protein	Bacteroides eggerthii	Bacteroides	$L_{1,2}$
1CQ3_A	132-171	Cytokine	Cowpox virus	Orthopoxvirus	$L_{4,2}$

Table 6: Protein chains with a two-sided lasso ($L_{i,j}$ type), i.e. with loops pierced by both tails, respectively i and j times. **In total 10 two-sided lassos have been identified in 10 proteins.**

PDB code	Loop range	Function (classification)	Organism Species	Organism Genus
1H30_A	283-570	Growth arrest spec.	Homo sapiens	Homo
1ZD0_A	48-131	Structural	Pyrococcus furiosus	Pyrococcus
2JH1_A	91-127	Cell adhesion	Toxoplasma gondii	Toxoplasma
2JH1_A	181-226	Cell adhesion	Toxoplasma gondii	Toxoplasma
2XJP_A	29-175	Cell adhesion	Saccharomyces cerevisiae	Saccharomyces
2XJP_A	176-263	Cell adhesion	Saccharomyces cerevisiae	Saccharomyces
2ZOU_A	44-128	Cell adhesion	Homo sapiens	Homo
3IAI_A	23-203	Lyase	Homo sapiens	Homo
3V5A_D	481-675	Metal binding protein	Bos taurus	Bos
3V83_C	474-665	Metal binding protein	Homo sapiens	Homo
3V83_C	137-331	Metal binding protein	Homo sapiens	Homo
4A3X_C	50-179	Cell adhesion	Candida glabrata	Nakaseomyces
4A3X_C	180-262	Cell adhesion	Candida glabrata	Nakaseomyces
4ASL_A	50-179	Cell adhesion	Candida glabrata	Nakaseomyces
4ASL_A	180-262	Cell adhesion	Candida glabrata	Nakaseomyces
4G7A_A	24-178	Lyase	Sulfurihydrogenibium sp. yo3aop1	Sulfurihydrogenibium
4HT2_A	22-202	Lyase	Homo sapiens	Homo
4KG7_A	54-123	Hydrolase	Mycobacterium smegmatis	Mycobacterium
4P1E_A	185-304	Transport	Escherichia fergusonii	Escherichia

Table 7: Protein chains with a supercoiling lasso (LS type), i.e. with loops pierced several times by one tail from the same direction. **In total 19 supercoiled loops have been identified, in 14 proteins.**

PDB code	Loop range	Lasso type	Function (classification)	Organism Species	Organism Genus	Chain type
1C01_A	11-64<br>23-49	L_1<br>L_1	Antimicrobial	Macadamia integrifolia	Macadamia	L_1L_1
1E4M_M	6-438<br>14-434	L_1<br>L_1	Hydrolase	Sinapis alba	Sinapis	L_1L_1
1FLC_A	126-174<br>196-238	L_1<br>L_1	Hydrolase	Influenza C virus	Influenzavirus C	L_1L_1
1HCN_B	23-72<br>26-110	L_1<br>L_1	Hormone	Homo sapiens	Homo	L_1L_1
1JY5_A	26-84<br>57-90	L_1<br>L_1	Hydrolase	Calystegia sepium	Calystegia	L_1L_1
1LKI_A	12-134<br>18-131	L_1<br>L_1	Cytokine	Mus musculus	Mus	L_1L_1
1Q25_A	81-111<br>385-419	L_1<br>L_1	Protein binding	Bos taurus	Bos	L_1L_1
1SGL_A	26-84<br>57-90	L_1<br>L_1	Hydrolase	Trichosanthes lepiniana	Trichosanthes	L_1L_1
1T61_A	53-108<br>164-222	L_1<br>L_1	Structural protein	Bos taurus	Bos	L_1L_1
1UDK_A	7-37<br>20-41	L_1<br>L_1	Unknown	Naja nigricollis	Naja	L_1L_1
1WKT_A	11-72<br>27-58	L_1<br>L_1	Toxin	Williopsis saturnus var. mrakii	Cyberlindnera	L_1L_1
2HCZ_X	42-70<br>73-140	L_1<br>L_1	Allergen 2	Zea mays	Zea	L_1L_1
2JTO_A	10-27<br>47-64	L_1<br>L_1	Hydrolase	Rhipicephalus bursa	Rhipicephalus	L_1L_1
2KQA_A	20-57<br>60-115	L_1<br>L_1	Toxin	Ceratocystis platani	Ceratocystis	L_1L_1
2PSP_A	8-35<br>58-84	L_1<br>L_1	Signaling	Sus scrofa	Sus	L_1L_1
2UUX_A	24-51<br>52-69	L_1<br>L_1	Inhibitor	Rhipicephalus appendiculatus	Rhipicephalus	L_1L_1
2ZK9_X	76-172<br>77-126	L_1<br>L_1	Hydrolase	Chryseobacterium proteolyticum	Chryseobacterium	L_1L_1
2ZX2_A	6-35<br>106-135	L_1<br>L_1	Immune system	Oncorhynchus keta	Oncorhynchus	L_1L_1
3SUK_A	39-76<br>79-138	L_1<br>L_1	Unknown	Moniliophthora perniciosa	Moniliophthora	L_1L_1
3SUM_A	43-80<br>83-145	L_1<br>L_1	Unknown	Moniliophthora perniciosa	Moniliophthora	L_1L_1
3U74_A	95-122<br>115-147	L_1<br>L_1	Hydrolase receptor	Homo sapiens	Homo	L_1L_1
4CYL_A	111-309<br>113-155	L_1<br>L_1	Cell adhesion	Caenorhabditis elegans	Caenorhabditis	L_1L_1
4HJ1_A	771-965<br>777-825	L_1<br>L_1	Viral	Rift valley fever virus	Phlebovirus	L_1L_1
4JP6_A	29-61<br>64-120	L_1<br>L_1	Unknown	Carica papaya	Carica	L_1L_1
4OIE_A	280-329<br>291-312	L_1<br>L_1	Viral	West nile virus	Flavivirus	L_1L_1

Continued on the next page

Table 8 – continued from the previous page

PDB code	Loops range	Lasso type	Function (classification)	Organism Species	Organism Genus	Chain type
1AOC_A	60-161<br>10-95	L_1<br>L_2	Coagulation factor	Tachypleus tridentatus	Tachypleus	L_1L_2
1ETE_A	44-127<br>93-132	L_1<br>L_2	Cytokine	Homo sapiens	Homo	L_1L_2
2OIZ_D	81-113<br>130-161	L_2<br>L_1	Oxidoreductase	Alcaligenes faecalis	Alcaligenes	L_2L_1
2Q9O_A	114-540<br>298-332	L_2<br>L_1	Oxidoreductase	Melanocarpus albomyces	Melanocarpus	L_2L_1
4H14_A	21-165<br>172-252	L_1<br>L_3	Viral	Bovine coronavirus	Betacoronavirus	L_1L_3
2CMZ_A	68-114<br>177-224	$LL_{1,1}$<br>L_1	Membrane	Vesicular stomatitis indiana virus	Vesiculovirus	$L_{1,1}L_1$
1CQ3_A	8-185<br>132-171	L_1<br>$L_{4,2}$	Antimicrobial	Macadamia integrifolia	Macadamia	$L_1L_{4,2}$
3V5A_N	425-647<br>481-675	L_1<br>LS	Metal binding	Bos taurus	Bos	L_1LS
3HHS_A	586-630<br>588-637	L_2<br>L_2	Oxidoreductase	Manduca sexta	Manduca	L_2L_2
2JH1_A	91-127<br>91-127	LS<br>LS	Cell adhesion	Toxoplasma gondii	Toxoplasma	$LSLS$
2XJP_A	29-175<br>176-263	LS<br>LS	Cell adhesion	Saccharomyces cerevisiae	Saccharomyces	$LSLS$
1DTV_A	18-62<br>19-43<br>22-58	L_1<br>L_1<br>L_1	Antimicrobial	Macadamia integrifolia	Macadamia	$L_1L_1L_1$
1WC2_A	30-69<br>65-178<br>72-157	L_1<br>L_1<br>L_1	Hydrolase	Mytilus edulis	Mytilus	$L_1L_1L_1$
2ENG_A	16-86<br>87-199<br>89-189	L_1<br>L_1<br>L_1	Hydrolase	Humicola insolens	Humicola	$L_1L_1L_1$
4ADI_A	37-242<br>49-287<br>51-130	L_2<br>L_1<br>L_1	Viral	Rubella virus	Rubivirus	$L_2L_1L_1$
2VGA_A	6-166<br>33-199<br>112-152	L_2<br>L_1<br>L_3	Viral	Vaccinia virus	Orthopoxvirus	$L_2L_1L_3$
3NKQ_A	148-194<br>156-350<br>413-801	L_1<br>L_2<br>$L_{1,2}$	Hydrolase	Mus musculus	Mus	$L_1L_2L_{1,2}$
1H30_A	283-570<br>444-470<br>562-609	LS<br>L_1<br>L_1	Growth arrest spec.	Homo sapiens	Homo	LSL_1L_1
2JD4_A	2686-2958<br>2845-2870<br>3024-3055	LS<br>L_1<br>L_1	Metal binding	Mus musculus	Mus	LSL_1L_1
4A3X_C	50-179<br>78-119<br>180-262	LS<br>$LS_{1,1}$<br>LS	Cell adhesion	Candida glabrata	Nakaseomyces	$LSLL_{1,1}LS$

Continued on the next page

Table 8 – continued from the previous page

PDB code	Loops range	Lasso type	Function (classification)	Organism Species	Organism Genus	Chain type
4ASL_A	50-179	LS	Cell adhesion	Candida glabrata	Nakaseomyces	$LSLL_{1,1}LS$
	78-119	$LS_{1,1}$				
	180-262	LS				
3V83_C	137-331	LS	Metal binding	Homo sapiens	Homo	LSL_1L_1LS
	402-674	L_1				
	418-637	L_1				
	474-665	LS				

Table 8: Protein chains with more than one pierced lasso in structure. **In total 16 different loop arrangements have been identified in 47 proteins.**

4 Posttranslational modifications

In order to reveal the possible function of the lasso motif the posstranslationally modified amino acids were selected in the set of topologically nontrivial structures. The analysis showed, that in the case of 4 protein chains the modified residue was located inside the pierced covalent loop (Tab. 9).

**PDB code**	**Loop range**	**Lasso loop type**	**Index of modified residue**	**Modified residue code**	**Modified residue name**	**Modified residue image**
3F5V_A	4-117	L_1	34	CSD	3-Sulfinoalanine	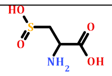
2VEC_A	10-204	L_1	122	CSO	S-Hydroxycysteine	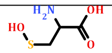
4IHZ_A	33-80	L_1	74	CSX	S-Oxy Cysteine	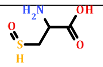
3MTW_A	172-213	L_1	211	KCX	Lysine Nϵ-Carboxylic Acid	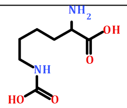

Table 9: The protein chains with posttranslational modifications found inside the pierced covalent loop.

In other 10 chains the modified residue was external to the covalent loop. As such residue can still influence the sequential, or spatial proximity of the piercing, we calculated the sequential distance between the modified residue and the closes piercing (Tab. 10).

**PDB code**	**Index of piercing residue**	**Index of modified residue**	**Distance**	**Modified residue code**	**Modified residue name**
4FDI_A	73	79	6	DDZ	3,3-Dihydroxy L-Alanine
2Q9O_A	92	98	6	OHI	3-(2-Oxo-2H-Imidazol-4-yl)- -L-Alanine
3QSD_A	127	324	197	074	[Propylamino-3-Hydroxy- -Butan-1,4-Dionyl]- -Isoleucyl-Proline
2HCZ_X	58	9	49	HYP	4-Hydroxyproline
1YG9_A	40	289	249	CSX	S-Oxy Cysteins
3QL6_A	179	198	19	SEP	Phosphoserine
2PSP_A	47	1	46	PCA	Pyroglutamic Acid
3KQ0_A	29	1	28	PCA	Pyroglutamic Acid
4GQR_A	63	1	62	PCA	Pyroglutamic Acid
4JP6_A	48	1	47	PCA	Pyroglutamic Acid

Table 10: The protein chains with modified residues, external to the pierced covalent loop. In the table the distance between the modified residue and the sequentially nearest piercing is given.

5 List of multimeric proteins

Multimeric proteins in study, for which at least one chain possess at least one pierced covalent loop (PDB codes with the chain names in parenthesis):
1AOC (A,B), 1AOZ (A,B), 1BCP (A,G), 1CQ3 (A,B), 1DEU (A,B), 1DOF (A,C,B,D), 1DP4 (A,C), 1DZK (A,B), 1EPA (A,B), 1ETE (A,C,B,D), 1F2L (A,C,B,D), 1FD3 (A,C,B,D), 1FJR (A,B), 1FLC (A,C,E), 1GXY (A,B), 1HC1 (A,C,B,E,D,F), 1I1J (A,B), 1I4U (A,B), 1IJV (A,B), 1IYB (A,B), 1JDP (A,B), 1JY5 (A,B), 1LE6 (A,C,B), 1M8A (A,B), 1N2Z (A,B), 1NF2 (A,C,B), 1NR4 (A,C,B,E,D,G,F,H), 1NSC (A,B), 1O7Z (A,B), 1OMZ (A,B), 1PZ7 (A,B), 1Q77 (A,B), 1QFT (A,B), 1RXD (C,B), 1SCF (A,B), 1T61 (A,C,B,E,D,F), 1TVX (A,C,B,D), 1TZP (A,B), 1UWC (A,B), 1WS8 (A,C,B,D), 1XTA (A,B), 1XTM (A,B), 1YRB (A,B), 1ZMI (A,C,B,D), 1ZMM (A,C,B,D), 1ZPU (A,C,B,E,D,F), 1ZXT (A,C,B,D), 2BB3 (A,B), 2BB6 (A,C,B,D), 2BGH (A,B), 2C1C (A,B), 2CKS (A,B), 2CMZ (A,C,B), 2D1G (A,B), 2D5W (A,B), 2DRE (A,C,B,D), 2E1V (A,B), 2F5X (A,C,B), 2GHV (C,E), 2GMF (A,B), 2GUM (A,C,B), 2HYX (A,C,B,D), 2JD4 (A,B), 2JIG (A,B), 2OIZ (H,D), 2OR7 (A,B), 2OYA (A,B), 2PMV (A,C,B,D), 2PSP (A,B), 2PT5 (A,C,B,D), 2Q9O (A,B), 2QKI (C,F), 2QN4 (A,B), 2RA4 (A,B), 2RA6 (A,C,B,D), 2W8X (A,B), 2W9X (A,B), 2WB9 (A,B), 2WY3 (B,D), 2XRC (A,C,B,D), 2Y8T (A,D), 2YAU (A,B), 2YG2 (A,B), 2Z4I (A,B), 2ZOU (A,B), 2ZX2 (A,B), 3A2E (A,D), 3AIH (A,B), 3B1B (A,B), 3BRN (A,B), 3BWK (A,B,D), 3CGU (A,B), 3CQN (A,B), 3EBW (A,B), 3EEQ (A,B), 3EQN (A,B), 3ETO (A,B), 3F5V (A,B), 3F95 (A,B), 3FIQ (A,B), 3FLP (A,C,B,E,D,G,F,I,H,K,J,M,L,N), 3FW3 (A,B), 3G7N (A,B), 3HEI (B,D,F,H,J,L,N,P), 3HHS (A,B), 3I26 (A,C,B,D), 3I5W (A,B), 3IAI (A,C,B,D), 3JXG (A,C,B,D), 3KGL (A,C,B,E,D,F), 3L49 (A,C,B,D), 3NGG (A,B), 3NK4 (A,B), 3NSW (C,B,D,G,F), 3ON9 (A,B), 3OP8 (A,B), 3PIM (A,B), 3PIV (A,B), 3Q31 (A,B), 3QTE (A,C,B,D), 3QW9 (A,B), 3RT4 (A,C,B,D), 3S8K (A,B), 3SAO (A,B), 3SUK (A,B), 3SUM (A,C,B,D), 3T94 (A,C,B,E,D,F), 3TC2 (A,C,B), 3U4Y (A,B), 3UTK (A,B), 3UYX (A,B), 3V83 (A,C,B,E,D,F), 3VUP (A,B), 3WKY (A,B), 3ZK4 (A,C,B), 3ZPX (A,B), 3ZXC (A,B), 4A7U (A,F), 4ADI (A,C,B), 4B7Q (A,C,B,D), 4BQD (A,B), 4CK4 (A,B), 4CMR (A,B), 4COF (A,C,B,E,D), 4ETR (A,B), 4F23 (A,C,B), 4FDI (A,B), 4FNK (A,C,E), 4G2U (A,B), 4G7A (A,B), 4GDI (A,C,B,E,D,F), 4GE1 (A,C,B,D), 4GQZ (A,C,B,D), 4GV5 (A,C,B), 4HJ1 (A,C,B,D), 4HT2 (A,C,B,D), 4IHZ (A,B), 4IO2 (A,B), 4JPH (A,C,B,D), 4K3Y (A,C,B,D), 4KNC (A,B), 4KYP (A,C,B,D), 4L3N (A,B), 4LB1 (A,B,E,D), 4LB7 (A,B,E,D), 4LBF (A,C,B,E,D,G,F,H), 4ODD (A,C,B), 4PMK (A,B), 4R2B (A,B).

6 Complex lasso classification based on CATH database

Lasso type	All	Mainly Alpha	Mainly Beta	Alpha Beta	Few secondary structures	Not classified
**Single,** L_1	296*	17<br>1ak0, 1ax8, 1bea<br>1dof, 1ete, 1gak<br>1jli, 1le6, 1lki<br>1mc2, 1n1f, ...	74<br>1ahl, 1aoc, 1aoz<br>1ata, 1b8w, 1bds<br>1c01, 1ccv, 1cq3<br>1d2s, 1d6b, ...	89<br>1ac5, 1aho, 1bcp<br>1cfe, 1cpy, 1dp4<br>1dtv, 1dys, 1e4m<br>1esc, 1fd3, ...	9<br>1bf0, 1g6x, 1kth<br>1tap, 1udk, 2j6d<br>2psp, 3ctk, 3ngg	107<br>1ijv, 1xtm, 1zmi<br>1zmm, 2bb6, 2cmz<br>2f5x, 2ghv, 2gum<br>2ikd, 2ike, ...
**Double,** L_2	46*	3<br>1ete, 2gmf, 2p3x	23<br>1aoc, 1br9, 1f2l<br>1g0y, 1gvz, 1hc1<br>1m8a, 1nr4, 1o7z<br>1rjt, 1tvx, ...	7<br>1kkh, 1omz, 1qfx<br>2ch9, 2yau, 3rt4<br>3tm0	—	14<br>1ypy, 2lt5, 2vga<br>2x97, 3f95, 3nkq<br>3nsw, 3tn2, 3zk4<br>4adi, 4hcs, ...
**Triple,** L_3	25*	1<br>3ql6	16<br>1bj7, 1dzk, 1epa<br>1kt6, 1lf7, 2l5p<br>2ra6, 2yg2, 3fiq<br>3kff, 3kq0, ...	4<br>1u3d, 2ehg, 3agn<br>3eeq	—	4<br>2vga, 4ck4, 4h14<br>4odd
**Sixfold,** L_6	1	—	—	—	—	1<br>4qi7
**Two-sided,** LL	10	—	1<br>1cq3	1<br>3om0	—	8<br>2cmz, 2d1g, 2dvz<br>2yhg, 3wa1, 4a3x<br>4asl, 4jgl
**Supercoiling,** LS	14	—	1<br>1h30	4<br>1zd0, 3iai, 3v5a<br>3v83	—	9<br>2jh1, 2xjp, 2zou<br>4a3x, 4asl, 4g7a<br>4ht2, 4kg7, 4p1e
Total	376**	20**	110**	103**	9**	134**

Table 11: Classification of complex lasso structures based on CATH data base.
* Few proteins are multidomain proteins, with various CATH classification. For those proteins the CATH number corresponding to the domain in which the piercings occur was chosen.
** 47 proteins posses more than one pierced loop (see Tab. 8 and therefore can be categorized into two lasso classes.

7 Examples of proteins with various lasso structures

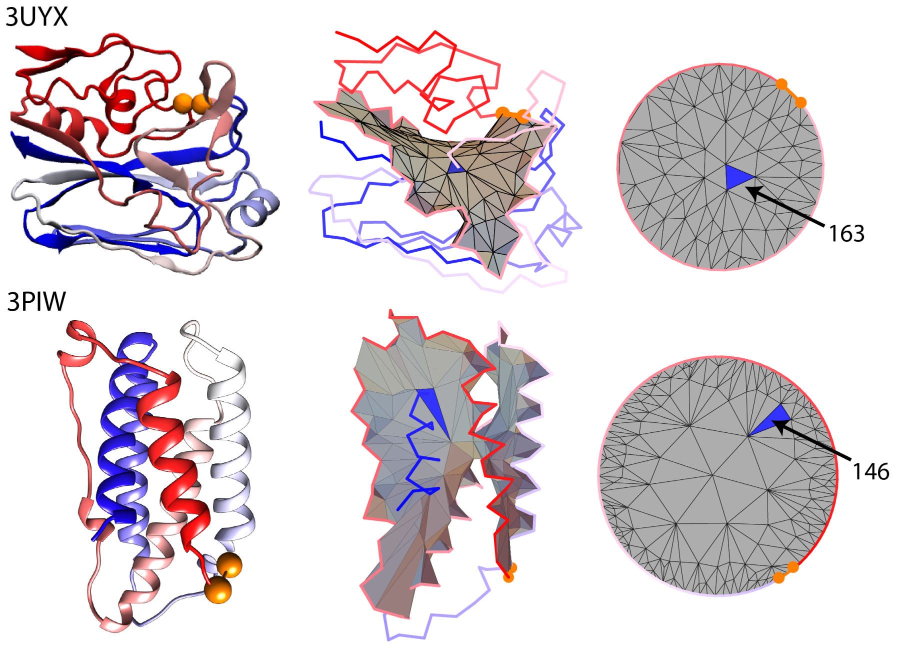

Figure 6: Proteins with L_1 topology consist of mainly beta strands (top row: protein with PDB code 3uyx) and mainly alpha helices (bottom row: protein with PDB code 3piw) based on CATH data base classification. Each row consists of the following panels: Left panel: cartoon representation of a given protein. Middle panel: triangulation of a minimal surface for this protein; the triangulated "soap bubble" surface, spanned on the covalent loop, is pierced once by a tail, through a triangle in blue; two cysteins and a cystein bond are shown in orange. Right panel: baricentric representation of a minimal triangulated surface for the same protein; two cysteins and a cystein bond comprise a part of the boundary and are shown in orange; blue triangle is pierced by a tail.

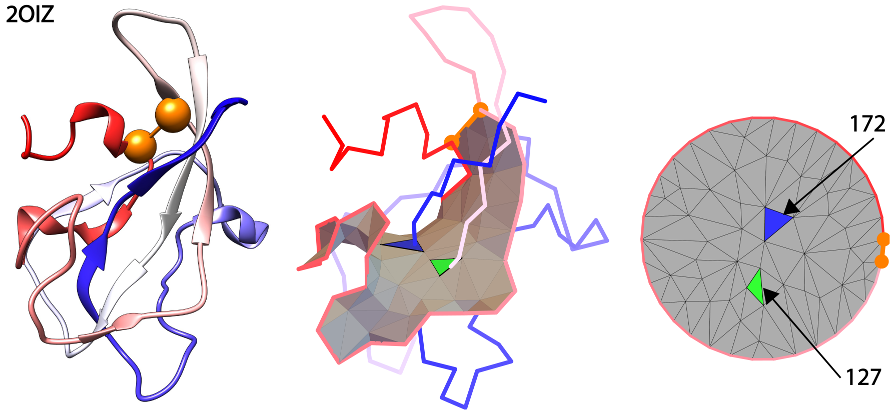

Figure 7: Protein with L_2 topology consist of mainly beta strands (top row: protein with PDB code 2oiz) based on CATH data base classification. Left panel: cartoon representation of a given protein. Middle panel: triangulation of a minimal surface for this protein; the triangulated "soap bubble" surface, spanned on the covalent loop, is pierced twice by a tail, through a triangle in blue and green; two cysteins and a cystein bond are shown in orange. Right panel: baricentric representation of a minimal triangulated surface for the same protein; two cysteins and a cystein bond comprise a part of the boundary and are shown in orange; blue and green triangles are pierced by a tail.

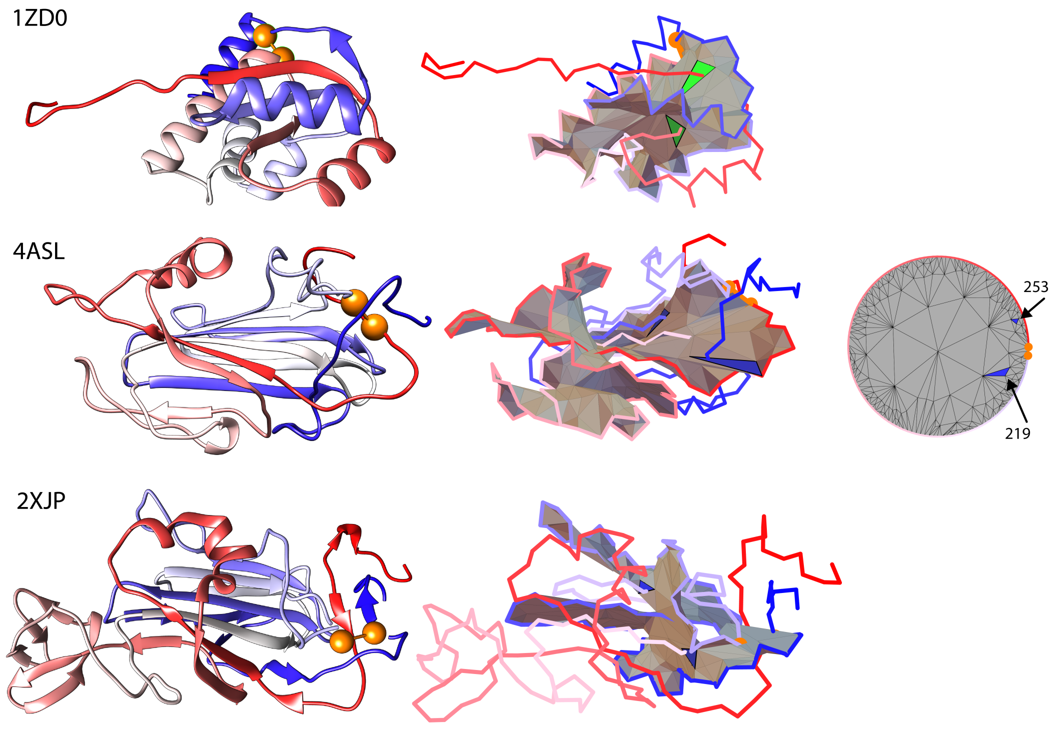

Figure 8: Proteins with LS motif. Proteins with LS topology consist of mainly alpha helices (top row: protein with PDB code 1zd0), mainly beta strands (middle row: protein with PDB code 4asl) and mainly beta strands (bottom row: protein with PDB code 2xjp) based on CATH data base classification. Each row consists of the following panels: Left panel: cartoon representation of a given protein. Middle panel: triangulation of a minimal surface for this protein; the triangulated "soap bubble" surface, spanned on the covalent loop, is pierced once by a tail, through triangles in green(top panel) or blue(middle and bottom panels); two cysteins and a cystein bond are shown in orange. Right panel: baricentric representation of a minimal triangulated surface for the same protein; two cysteins and a cystein bond comprise a part of the boundary and are shown in orange; green triangles are pierced by a tail two times in the same direction.

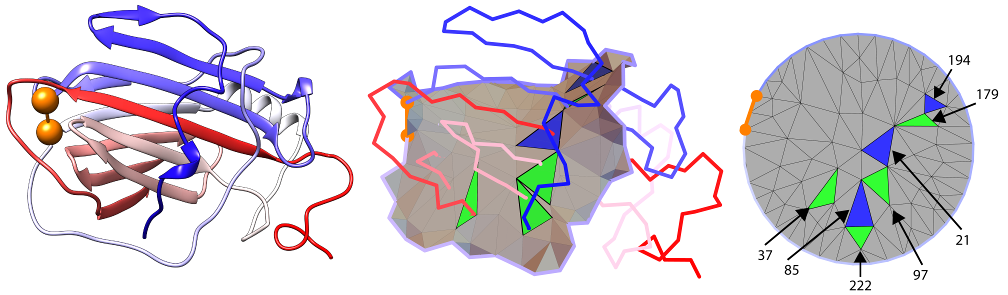

Figure 9: Protein 1cq3 with LL motif. Example of protein with the most complicated LL topology which consist of mainly beta strands (PDB code 2oiz) based on CATH data base classification. Left panel: cartoon representation of a given protein. Middle panel: triangulation of a minimal surface for this protein; the triangulated "soap bubble" surface, spanned on the covalent loop, is pierced four times by the N-terminal tail and three times by the C-terminal through a triangles in blue and green; two cysteins and a cystein bond are shown in orange. Right panel: baricentric representation of a minimal triangulated surface for the same protein; two cysteins and a cystein bond comprise a part of the boundary and are shown in orange; blue and green triangles are pierced by the N-terminal and C-terminal tails.

8 Analysis of proteins with small covalent loops

In the case of mini-proteins (sec. 9) the piercing chain fragment is usually stabilized by bulky residues before and after piercing. Therefore we analyzed all lasso proteins with pierced loop comprising maximally 30 residues and checked if there is any bulky residue (TRP, TYR, PHE, MET, ARG, HIS, LYS, LEU) in the range of 5 residues from piercing. The results are contained in Tab. 12.

**PDB code**	**Loop range**	**Loop size**	**Loop type**	**Piercing residue index**	**Bulky residues**
1B8W_A	16-32	17	L_1	38	Arg41
1BDS_A	6-32	27	L_1	39	Trp35, His43
1BF0_A	32-53	22	L_1	23	Phe20, Phe25
1C01_A	23-49	27	L_1	71	Trp69, Phe73
1D6B_A	16-32	17	L_1	38	Arg35, Tyr42
1DTV_A	19-43	25	L_1	9	Tyr12
1F2L_A	8-34	27	L_2	39<br>50	Arg37, Leu41<br>Phe49, Lys54
1FD3_A	15-30	16	L_1	36	Leu32, Lys39
1G6X_A	30-51	22	L_1	21	Arg20, Phe23
1IJV_A	12-27	16	L_1	33	Lys31, Lys36
1KJ6_A	18-33	16	L_1	39	Arg38, Arg42
1KTH_A	30-51	22	L_1	21	Lys20, Tyr22
1M4L_A	138-161	24	L_1	165	Lys168
1M8A_A	6-32	27	L_2	37<br>48	Phe39, Leu45<br>Phe49, Lys52
1NR4_A	10-34	25	L_2	39<br>50	Arg36, Phr38<br>Phe47
1O7Z_A	9-36	28	L_2	41<br>53	Arg38, Lys46<br>Arg52, Lys54
1OK0_A	45-73	29	L_1	33	Lys34
1RJT_A	9-36	28	L_2	41<br>52	Lys38, Leu45<br>Lys49, Leu54
1SHI_A	5-33	29	L_1	44	Lys46
1TAP_A	33-55	23	L_1	24	Arg23, Tyr25
1TVX_A	25-51	27	L_2	56<br>67	Leu60<br>Lys65, Leu68
1UDK_A	20-41	22	L_1	44	Phe43
1WQK_A	6-30	25	L_1	37	Leu34, Tyr39
1ZMI_A	3-18	16	L_1	26	Trp25, Phe27
1ZMM_A	4-19	16	L_1	27	Phe26, Tyr28
1ZXT_A	12-36	25	L_2	41<br>52	Lys38, Leu43<br>Arg49, Lys57
2HCZ_X	42-70	29	L_1	94	Tyr92, Tyr98
2HDL_A	3-29	27	L_2	34<br>50	Lys32, Lys39<br>His49, Leu51
2J6D_A	35-56	26	L_1	34	Arg33, Tyr35
2JD4_B	2845-2870	26	L_1	2699	Tyr2694, Phe2701
2JR3_A	16-32	17	L_1	37	Phe36, Arg40
2JTO_A	10-27<br>47-64	18<br>18	L_1<br>L_1	30<br>69	Leu34<br>Lys66, Lys74

Continued on the next page

Table 12 – continued from the previous page

PDB code	Loop range	Loop size	Loop type	Piercing residue index	Bulky residues
2KER_A	43-70	28	L_1	31	Tyr32
2LVX_A	408-437	30	L_1	444	Lys442
2MJK_A	12-28	17	L_1	31	Lys29, Leu34
2MN3_A	16-30	15	L_1	35	Arg32, Phe39
2PSP_A	8-35	28	L_1	47	Trp45, Lys48
	58-84	27	L_1	95	Tyr94, Phe96
2RA4_A	11-35	25	L_2	40	Lys38, Phe42
				51	Lys48, Lys55
2RNG_A	52-70	19	L_1	74	Tyr73, Arg77
2UUX_A	24-51	28	L_1	58	Tyr55, Tyr59
	52-69	18	L_1	45	Arg44, Ty46
2W8X_A	51-69	19	L_1	42	Leu41, His43
2XFD_A	90-101	12	L_1	83	Trp85
2ZX2_A	6-35	30	L_1	88	Trp87, Lys91
	106-135	30	L_1	188	Tyr187, Tyr192
3GV3_A	9-34	26	L_2	38	Leu36, Arg41
				50	Arg47, Lys54
3I5W_A	5-20	16	L_1	28	Tyr27, Leu29
3NGG_A	10-35	26	L_1	41	Lys40, Arg44
3OZP_A	36-55	20	L_1	28	Trp27, Trp29
3QTE_A	6-20	15	L_1	28	Trp27, Phe29
3TN2_A	11-35	25	L_2	40	Phe42
				51	Lys48
4BQD_A	51-72	22	L_1	42	Arg41, Phe43
4GV5_A	11-30	20	L_1	35	Trp34, Lys38
4HCS_A	15-40	26	L_2	44	Lys42, Leu45
				55	Lys54
4JJO_A	23-48	26	L_1	65	Arg62
4KYP_A	43-69	27	L_1	4	Lys2, Tyr5
4LB1_A	4-19	16	L_1	27	Trp26
4LBF_A	4-19	16	L_1	27	Trp26, Phe28
4OIE_A	291-312	22	L_1	335	Tyr331, Arg336

Table 12: The protein chains with small covalent loops (comprasing maximally 30 residues) with potentially blocking bulky residues. For each piercing the closest bulky residue before and after piercing (if they exist) is given.

9 Mini-proteins

The column entitled ”Lasso stabilization amino acids” contains the information, which amino acids occur before, and which after the plug. This information can be directly compared with the crossing position given in the column ”Surface piercing bond”. It is worth mentioning, that our method in several cases agrees with the experimental results. In case of Xanthomycin, BI-32169 and Sviceucin our method predicts exact position of the surface crossing, which is inside the region determined by experimental data between the closest bulky aminoacids. Only in case of Astexin 1(23) our calculated data differ from the experimental slightly. This implies, that our analysis can predict also the aminoacids stabilizing the topology.

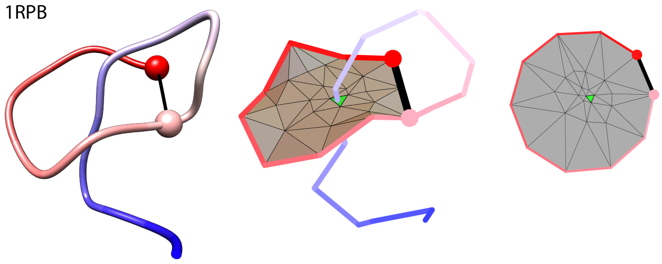

Figure 10: Protein with L_1 topology or lasso topology identified in mini-protein (PDB code 1rpb). Left panel: cartoon representation of a given protein. Middle panel: triangulation of a minimal surface for this protein; the triangulated “soap bubble” surface, spanned on the covalent loop, is pierced once by a tail, through a triangle in green; Cys (number one, red boal) and Asp (number 9, pink) and amide bond between them is shown in black. Right panel: baricentric representation of a minimal triangulated surface for the same protein; an amide bond comprise a part of the boundary is shown in black; green triangle is pierced by a tail.

	Peptide	PDB id	Peptide length (aa)	Id of atoms forming bond	Lasso stabilization amino acids[a]	Surface piercing bond[a]
Class I	Aborycin RP71955	1rpb	21	Cys1-Asp9	2 S-S bridges Cys1-Cys13 Cys7-Cys19	Tyr15-Ala16
Class II	Astexin 1(23)	2lti	23	Gly1-Asp9	Tyr14/**Phe15**	**Glu17-Ser18**
	Astexin 1(19)[c]	2m37	19	Gly1-Asp9	Tyr14/**Phe15**	Tyr14-Phe15
	Astexin 3	2m8f	24	Gly1-Asp9	Tyr15/**Trp16**	Tyr15-Trp16
	Caulosegnin I	2lx6	19	Gly1-Glu8	Arg15/**Glu16**	Arg15-Glu16
	Microcin J25	1pp5	21	Gly1-Glu8	Phe19/**Tyr20**	Phe19-Tyr20*
	Microcin J25[d]	1s7p	21	Gly1-Glu8	—	Phe19-Tyr20*
	Streptomonomicin STM	2mw3	21	Ser1-Asp9	—	Pro14-Ala15
	Caulonodin V	2mlj	18	Ser1-Glu9	—	Tyr16-Trp17*
	Xanthomonin I	2mfv	14[e]	Gly1-Glu7	Ile9/**Phe12**	**Gly10-Gly11**
	Xanthomonin II	4nag	16[e]	Gly1-Glu7	Met9/**Ile12**	**Gly10-Gly11**
Class III	BI-32169 The glucagon receptor antagonist	3njw	19	Gly1-Asp9	1 S-S bridge Cys6-Cys19/ *Trp13*/**Trp17**	**Asn14-Thr15**
Class IV	Sviceucin	2ls1	20	Cys1-Asp9	2 S-S bridges Cys1-Cys13 Cys7-Cys19/ **Trp17**	**Thr15-Ala16**

Table 13: Lasso peptides - all have L1 topology according to our notation.
[a] According to [5]. Stabilization can occur by two bulky amino acids or by the presence of cysteine bonds. In case of bulky amino acids residue after pluging through the loop is in bold character whereas the residue before is not. Residues that have been hypothesized to stabilize the topology, but have not been identified by structural analysis or mutagenesis are in italics. For some proteins data of stabilizing residues are missing.
[b] According to introduced method. Entries which differ with entries in "Lasso stabilization amino acids" column are in bold.
[c] Astexin 1 was produced from the wild type strain and by heterologous expression under a 23-amino acid form accompanied by truncated forms, among which Astexin 1(19) was characterized.
[d] Microcin J25 protein with PDB id 1s7p has exactly the same amino acid sequence as Microcin J25 with 1pp5 protein, but according to crystal structure the chain of 1pp5 is splited into two separate chains in 1s7p.
[e] Xanthomonins I, II are of lenght 20 amino acids, but the structure deposited in PDB are truncated to the number of residues given in table.
* These intersections are shallow, i.e. they are close to the end of a tail or to the ring (in the distance less or equal 3 aa), so would not be counted as complex lasso in our analysis.

10 Structural alignment of proteins with L_6 lasso type

The L_6 lasso protein (cellobiose dehydrogenase) with PDB code 4qi7 is a two domain protein with only one homolog (PDB code 4qi6). One of the bridge forming cysteines is located in the linker joining two domains. This part is missing in the homolog, causing its trivial lasso type. Both structures (lasso containing domain with linker) are aligned in the Fig 11.

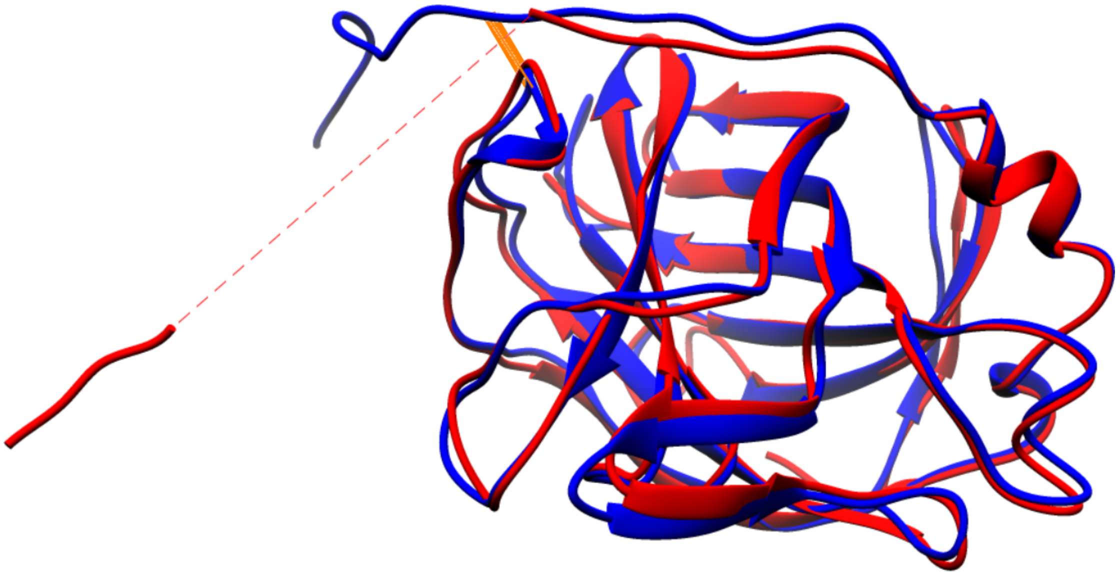

Figure 11: Structural alignment of cellobiose dehydrogenase with L_6 lasso type (blue structure, PDB code 4qi7) with its homolog (red structure, PDB code 4qi6). The covalent loop closing bridge is depicted as orange stripe. The missing fragment is denoted as a dashed line. To facilitate view, only one of two domains in each proteins is displayed.

References

[1] Chen W, Cai Y, Zheng J (2008) Constructing triangular meshes of minimal area. *Computer-Aided Design and Applications 5* 508-518.

[2] Pettersen, E.F., Goddard, T.D., Huang, C.C., Couch, G.S., Greenblatt, D.M., Meng, E.C., and Ferrin, T.E. (2004) UCSF Chimera - A Visualization System for Exploratory Research and Analysis. *J. Comput. Chem.* 25(13):1605-1612

[3] William T. Tutte, W.T. (1963) How to draw a graph. *Proc. London Math. Society* 13(52):743-768

[4] Jamroz M, Niemyska W, Rawdon EJ, Stasiak A, Millett KC, Sulkowski P, Sulkowska JI (2014) KnotProt: a database of proteins with knots and slipknots. *Nucl. Acids Res.* D306-D314

[5] Li Y, Zirah S, Rebuffat S (2015) Lasso Peptides. Bacterial Strategies to Make and Maintain Bioactive Entangled Scaffolds. *SpringerBriefs in Microbiology*
